# Sphingosine 1-Phosphate Induces Cyclooxygenase-2/Prostaglandin E_2_ Expression via PKCα-dependent Mitogen-Activated Protein Kinases and NF-κB Cascade in Human Cardiac Fibroblasts

**DOI:** 10.3389/fphar.2020.569802

**Published:** 2020-10-30

**Authors:** Chien-Chung Yang, Li-Der Hsiao, Mei-Hsiu Su, Chuen-Mao Yang

**Affiliations:** ^1^ Department of Traditional Chinese Medicine, Chang Gung Memorial Hospital at Tao-Yuan, Tao-Yuan, Taiwan; ^2^ School of Traditional Chinese Medicine, College of Medicine, Chang Gung University, Tao-Yuan, Taiwan; ^3^ Department of Pharmacology, College of Medicine, China Medical University, Taichung, Taiwan; ^4^ Department of Post-Baccalaureate Veterinary Medicine, College of Medical and Health Science, Asia University, Wufeng, Taichung, Taiwan

**Keywords:** human cardiac fibroblasts, NF-κB, mitogen-activated protein kinases, PKCα, cyclooxygenase-2, sphingosine 1-phosphate

## Abstract

In the regions of tissue injuries and inflammatory diseases, sphingosine 1-phosphate (S1P), a proinflammatory mediator, is increased. S1P may induce the upregulation of cyclooxygenase-2 (COX-2)/prostaglandin E_2_ (PGE_2_) system in various types of cells to exacerbate heart inflammation. However, the detailed molecular mechanisms by which S1P induces COX-2 expression in human cardiac fibroblasts (HCFs) remain unknown. HCFs were incubated with S1P and analyzed by Western blotting, real time-Polymerase chain reaction (RT-PCR), and immunofluorescent staining. Our results indicated that S1P activated S1PR_1/3_-dependent transcriptional activity to induce COX-2 expression and PGE_2_ production. S1P recruited and activated PTX-sensitive G_i_ or -insensitive G_q_ protein-coupled S1PR and then stimulated PKCα-dependent phosphorylation of p42/p44 MAPK, p38 MAPK, and JNK1/2, leading to activating transcription factor NF-κB. Moreover, S1P-activated NF-κB was translocated into the nucleus and bound to its corresponding binding sites on COX-2 promoters determined by chromatin immunoprecipitation (ChIP) and promoter-reporter assays, thereby turning on COX-2 gene transcription associated with PGE_2_ production in HCFs. These results concluded that in HCFs, activation of NF-κB by PKCα-mediated MAPK cascades was essential for S1P-induced up-regulation of the COX-2/PGE_2_ system. Understanding the mechanisms of COX-2 expression and PGE_2_ production regulated by the S1P/S1PRs system on cardiac fibroblasts may provide rationally therapeutic interventions for heart injury or inflammatory diseases.

## Introduction

Sphingosine 1-phosphate (S1P) has been characterized as a potent bioactive sphingolipid molecule that regulates a wide range of physiological and pathological functions, including inflammation, angiogenesis, endothelial barrier function, lymphocyte egress, apoptosis, and proliferation (Proia and Hla, 2015; Pyne et al., 2016; Vogt and Stark, 2017). Large amounts of S1P have been identified in the synovial fluid of patients with rheumatoid arthritis (Kitano et al., 2006), the serum of lupus patients with juvenile-onset (Watson et al., 2012), and the bronchoalveolar lavage of asthmatics (Ammit et al., 2001), which enhance the inflammatory responses through upregulation of adhesion molecules and recruitment of inflammatory cells and lymphocytes (Florey and Haskard, 2009; Milara et al., 2009). More recently, the elevated levels of S1P and sphingomyelin have been detected in the plasma of patients, which are associated with left ventricular function and clinical signs of heart failure (Polzin et al., 2017), and prevalence and severity of coronary artery disease and myocardial infarction (Levkau, 2015). Cardiac S1P has greatly increased post-myocardial infarction leading to cardiac inflammation, dysfunction, and remodeling (Zhang et al., 2016). S1P-triggered most of the cellular functions are mediated through interaction with one of the five G-protein coupled receptors (S1PR_1–5_) leading to activation of downstream signaling mechanisms associated with the pathogenesis of various diseases (Alvarez et al., 2007; Maceyka et al., 2012). Furthermore, S1PR_1-3_ but not S1PR_4/5_ are expressed on the cardiovascular systems of rat and human (Ishii et al., 2004; Landeen et al., 2007; Ahmed et al., 2017) detected by Real Time-Polymerase chain reaction (RT-PCR). Cardiac fibroblasts are one of main cellular constituents of the heart and play numerous roles in cardiac functions including proliferation, migration, secretion of growth factors and cytokines, and inflammation (Porter and Turner, 2009). Therefore, S1P may play an important role in regulation of cardiovascular functions and can be a therapeutically target in inflammatory heart disorders.

Cyclooxygenase (COX) catalyzes the rate-limiting reaction in the synthesis of prostaglandins (PGs) in various tissues, which consists of two isoforms of COX, in terms as COX-1 and COX-2. COX-1 is constitutively expressed and maintains the homeostasis of physiological functions (O’Neill and Ford-Hutchinson, 1993). In contrast, COX-2 is absent or present at a low level and could be induced by various factors such as hormones, neurotransmitters, cytokines, and mitogens and initiates the synthesis of PGs during various inflammatory conditions (Turini and DuBois, 2002). Therefore, COX-2 plays a key role in the conversion of arachidonic acid into PGH_2_ leading to the release of proinflammatory PGs, such as PGE_2_ in various types of cells and tissues. Previously, several studies have demonstrated that S1P could induce COX-2 expression accompanied by the release of PGE_2_, and leading to inflammatory responses in various models of cells and organs (Kim et al., 2003; Hsieh et al., 2006; Ohama et al., 2008; Volzke et al., 2014; Hsu et al., 2015; Rumzhum et al., 2016). In addition, the expression of COX-2 has been confirmed by an immunohistochemical staining in the inflammatory tissues such as synovial macrophages and vascular endothelial cells from patients diagnosed with arthritis and atherosclerosis. In clinics, the selective COX-2 inhibitors are widely used for the treatment of these inflammatory diseases (Williams et al., 1999; Korotkova et al., 2005). Moreover, silencing of COX-2 gene in mice also markedly attenuates the LPS-mediated inflammation (Ejima et al., 2003). In the heart, rapid induction of COX-2 in cardiomyocytes has been demonstrated in the experimental model of chronic heart failure (Zidar et al., 2007). Therefore, COX-2 may play a key role in the development of heart inflammatory diseases.

In cardiac ﬁbroblasts, S1P regulates a wide range of physiological functions such as the proliferation of the cells, production of collagen, and cell migration (Lampasso et al., 2002; Kim et al., 2003; Pitson et al., 2003; Thompson et al., 2006; Means and Brown, 2009). The major mechanisms by which S1P activates responses are mediated through S1PR_3_ coupling G_q_ proteins to activate phospholipase C (PLC)-β, hydrolyze phosphoinositide (PI), and result in the formation of inositol trisphosphate (IP_3_) and diacylglycerol, ultimately leading to Ca^2+^ increase and protein kinase C (PKC) activation, respectively (Takuwa et al., 2008). Activation of a G_i_ protein-coupled S1PR_1/3_ has an inhibitory effect on adenylyl cyclase activity (Takuwa et al., 2008). These physiological actions are mediated through the activation of several intracellular protein kinases pathways including PKCs and mitogen-activated protein kinases (MAPKs) (Pyne and Pyne, 2000; Tanimoto et al., 2004; Singleton et al., 2005). MAPKs consist of three subfamilies including p38 MAPK, p42/p44 MAPK, and c-Jun N-terminal kinase (JNK1/2) (Marshall, 1994). PKC isoforms have also been shown to be the major mediators that activate MAPK cascade. Previously, activation of MAPKs and related transcription factors are necessary for the up-regulation of COX-2 (Pratt et al., 2003). S1P has been shown to induce COX-2 expression in various types of cells (Kim et al., 2003; Hsieh et al., 2006; Ohama et al., 2008; Volzke et al., 2014; Hsu et al., 2015; Rumzhum et al., 2016), accompanied by PGE_2_ biosynthesis which might participate in several inflammatory responses (Candela et al., 1991; Davaille et al., 2000). S1P has also been shown to activate NF-κB in many cell types (Siehler et al., 2001; Hsieh et al., 2006) and regulate the expression of inflammatory genes (Barnes and Karin, 1997). Our previous researches indicated that several GPCR agonists (e.g., S1P, bradykinin, and thrombin) stimulate MAPKs and NF-κB activation leading to COX-2 expression in various types of cells (Hsieh et al., 2006; Hsieh et al., 2007; Hsieh et al., 2008; Hsu et al., 2015). Therefore, COX-2 expression could be regulated by a series of PKCs, MAPKs, and transcription factors, including NF-κB (Barnes and Karin, 1997; Tanabe and Tohnai, 2002). However, the molecular and signaling mechanisms underlying S1P-induced COX-2 expression are not completely defined in human cardiac fibroblasts (HCFs).

Although several proinflammatory mediators have been shown to play critical roles in inflammation due to the up-regulation of NF-κB-dependent genes such as COX-2 (Barnes and Karin, 1997; Hsieh et al., 2008), the detail mechanisms by which S1P induced COX-2 expression and PGE_2_ release were investigated in HCFs by using pharmacological inhibitors and gene silencing approaches. These findings indicated that S1P-induced COX-2 expression is mediated through transcription and translation, which are via coupling to both G_i_ and G_q_ protein S1PR_1/3_, activation of PKCα-dependent p42/p44 MAPK, p38 MAPK, JNK1/2, and NF-κB signaling pathways, leading to PGE_2_ production in HCFs. These results deliver new insights into the mechanisms underlying S1P effects and may offer therapeutic value in heart inflammatory disorders.

## Materials and Methods

### Materials

DMEM/F-12 medium, fetal bovine serum (FBS), TRIzol reagent and PLUS-Lipofectamine, and p38 siRNA (MAPK14HSS102352) were purchased from Invitrogen (Carlsbad, CA, United States). Hybond C membrane, enhanced chemiluminescence (ECL) and Western blotting detection system were purchased from GE Healthcare Biosciences (Buckinghamshire, United Kingdom). COX-2 antibody was from Abcam (Cambridge, United Kingdom). PhosphoPlus p42/p44 MAPK (#9101), p38 MAPK (#9211), JNK1/2 (#4668), p65 NF-κB (#3031), and I-κB (#2859) antibodies were from Cell Signaling (Danvers, MA, United States). S1PR1 (sc-48356), S1PR3 (sc-30024), G_αq/11_ (sc-392), G_αi-3_ (sc-262), PKCα (sc-208), p42 (sc-154), p38 (sc-33688), JNK1 (sc-474), and p65 NF-κB (sc-7151) antibodies were from Santa Cruz (Santa Cruz, CA, United States). Sphingosine 1-phosphate (S1P), W123, JTE-013, and CAY10444 were from Cayman (Ann Arbor, MI, United States). GPA2A, pertussis toxin (PTX), Gö6976, PD98059, SB202190, SP600125, and helenalin were from Biomol (Plymouth Meeting, PA, United States). Glyceraldehyde 3-phosphate dehydrogenase (GAPDH) antibody was purchased from Biogenesis (Bournemouth, United Kingdom). Bicinchoninic acid (BCA) protein assay kit was purchased from Pierce (Rockford, IL, United States). Enzymes, siRNAs for S1PR1 (SASI_Hs01_00245812, NM_001400), S1PR3 (SASI_Hs01_00011116, NM_005226), G_q_ (SASI_Hs01_00148143, NM_001002911), G_i_ (SASI_Hs01_00187238, NM_02278), PKCα (SASI_Hs01_00018817, NM_002737), p42 (SASI_Hs01_00058601, NM_004364), JNK1 (SASI_Hs02_00319556, NM_001098625), and p65 NF-κB (SASI_Hs01_00171091, NM_021975) and other chemicals were from Sigma (St. Louis, MO, United States). SDS-PAGE supplies were from MDBio Inc (Taipei, Taiwan).

### Cell Culture and Treatment

HCFs were isolated from the human heart obtained from ScienCell Research Laboratories (San Diego, CA, United States). DMEM/F-12 containing 10% (v/v) FBS, 2 mM glutamine and antibiotics (100 U/ml penicillin G, 100 μg/ml streptomycin, and 250 ng/ml fungizone) was used for cell culture, and these cells were grown at 37°C with a humidified 5% CO_2_ atmosphere. When these cells reached confluence, cells were treated with 0.05% (w/v) trypsin/0.53 mM EDTA for 1 min at 37°C. The cell suspension was diluted with DMEM/F-12 containing 10% FBS and 2 mM glutamine to a concentration of 2 cells/ml × 10^5^ cells/ml. The cells were plated onto 12-well culture plates and made quiescent at confluence by incubation in serum-free DMEM/F-12 containing 0.1% BSA and 2 mM glutamine for 24 h, and then incubated with S1P at 37°C for the indicated time intervals as the experimental protocol. When the inhibitors were used, cells were pretreated with the inhibitor for 1 h, and then incubated with S1P. Experiments were performed using cells from passages 4 to 7.

### Preparation of Cell Extracts and Western Blot Analysis

Growth-arrested HCFs were incubated with S1P at 37°C for the indicated time intervals. To yield the whole-cell extract, the cells were washed with ice-cold PBS, scraped, and collected by centrifugation at 45,000 × g for 1 h at 4°C, as previously described (Hsieh et al., 2006). Samples were denatured and subjected to SDS-PAGE using a 10% (w/v) running gel; then proteins were transferred to nitrocellulose membrane. Membranes were incubated with a phospho-p42/p44 MAPK, phospho-p38 MAPK, phospho-JNK1/2, phospho-p65 NF-κB, phospho-I-κB, S1PR1, S1PR3, G_q_, G_i_, PKCα, p38α, JNK1, p42, p65, or GAPDH antibody overnight. TTBS washed the membranes with four times for 5 min each, then the membranes were incubated with an anti-rabbit horseradish peroxidase antibody of a 1:2000 dilution for 1 h. ECL reagents were used to detect the immunoreactive bands, quantified by UN-SCAN-IT gel version 6.1 (Orem, Utah, United States). The densitometry of the specific band was normalized to GAPDH. Each experiment was performed in three individual experiments.

### Total Ribonucleic Acid Extraction and Polymerase Chain Reaction/Real Time-Polymerase Chain Reaction Analysis

According to the protocol of the manufacturer, total RNA was isolated from HCFs treated with S1P for the indicated time in 10 cm culture dishes using TRIzol. To produce a template for PCR amplification, the cDNA obtained from 0.5 μg total RNA was prepared, as previously described (Tung et al., 2010). Oligonucleotide primers were designed based on Genbank entries for human COX-2, S1PR (types 1–3), and *β*-actin. The following primers were used for amplification reaction

#### COX-2

5′-AAA​ACC​GTG​GGG​AAT​GTA​TGA​GC-3′ (forward primer);

5′-GAT​GGG​TGA​AGT​GCT​GGG​GAA​AG-3′ (reverse primer);

#### S1PR1

5′-GTG​TAG​ACC​CAG​AGT​CCT​GCG-3′ (forward primer);

5′-AGC​TTT​TCC​TTG​GCT​GGA​GAG-3′ (reverse primer);

#### S1PR2

5′-GGC​CTA​GCC​AGT​GCT​CAG​C-3′ (forward primer);

5′-CCT​TGG​TGT​AAT​TGT​AGT​GTT​CCA​GA-3′ (reverse primer);

#### S1PR3

5′-GGA​GCC​CCT​AGA​CGG​GAG​T-3′ (forward primer);

5′-CCG​ACT​GCG​GGA​AGA​GTG​T-3′ (reverse primer);

#### **β**-actin

5′-GAA​CCC​TAA​GGC​CAA​CCG​TG-3′ (forward primer);

5′-TGG​CAT​AGA​GGT​CTT​TAC​GG-3′ (forward primer).

PCR mixes contain 1.25 mM of each dNTP, 10 μl of 5× PCR buffer, 100 pmol of each forward and reverse primer, and 2.5 units of Taq polymerase (Takara, Shiga, Japan). Fifty microliter was the final reaction volume. The program for COX-2 amplification was performed in 25 cycles as follows: at 94°C for 20 s, 60°C for 40 s, and 72°C for 40 s. After the last cycle, all samples were incubated for an additional 10 min at 72°C. 2% agarose 1× TAE gel containing ethidium bromide was used to analyze the PCR fragments and their sizes were compared to a molecular weight marker. Amplification of *β*-actin was performed in parallel as a relatively invariant internal reference RNA, and cDNA amounts were standardized to equivalent *β*-actin mRNA levels. These primer sets recognized only the genes of interest as indicated by amplification of a specific single band of the expected size (500 bp for COX-2, 482 bp for S1PR1, 611 bp for S1PR2, 724 bp for S1PR3, and 514 bp for *β*-actin) and direct sequence analysis of the PCR product.

Real time-PCR was performed with the TaqMan gene expression assay system, using primers and probe mixes for COX-2 and endogenous GAPDH control genes as previously described (Tung et al., 2010). PCRs were performed using a 7,500 Real Time-PCR System (Applied Biosystems, Foster City, CA, United States). Relative gene expression was determined by the ΔΔCt method, where Ct meant the threshold cycle.

### Plasmid Construction, Transfection, and Luciferase Reporter Gene Assays

For the construction of the κB-Luc and COX-2-Luc plasmids, a human COX-2 promoter located within a region spanning from −483 to +37 bp was cloned into the pGL3-basic vector (Promega, Madison, WI, United States). A series of point mutations were introduced into the NF-κB binding site of COX-2 promoter by mismatched primer mutation PCR as previously described (Tung et al., 2010). The plasmids were prepared by using QIAGEN plasmid DNA preparation kits and transfected into HCFs using the Lipofectamine reagent according to the instructions of the manufacturer. According to the manufacturer’s instructions, a luciferase assay system (Promega, Madison, WI, United States) was used to measure the COX-2-Luc activity. *β*-galactosidase activity standardized firefly luciferase activities.

### Transient Transfection With Small interfering Ribonucleic Acids

The SMARTpool-siRNAs of scramble, S1PR1, S1PR3, G_q_, G_i_, PKCα, p42, p38α, JNK1, and p65 were from Sigma (St. Louis, MO, United States). HCFs cells were plated at 3 cells/ml × 10^5^ cells/ml (1 ml per well) in 12-well culture plates for 24 h, reaching about 80% confluence. Cells were incubated with 0.4 ml of serum-free DMEM/F-12 medium each well after washed once with PBS and once with serum-free DMEM/F-12. The siRNA was prepared and transiently transfected using Lipofectamine 2000 transfection reagent according to the instructions of the manufacturer.

### Isolation of Subcellular Fractions

After treatment, harvested cells were sonicated for 5 s at output 1.5 with a sonicator (Misonix, Farmingdale, NY, United States). Then, the cell lysates were centrifuged at 8,000 rpm for 15 min at 4°C. Both the pellet and supernatant were collected, respectively. The pellet was saved as the nuclear fraction. The supernatant was saved for further centrifugation at 14,000 rpm for 60 min at 4°C. The pellet and the supernatant were respectively collected as membrane fraction and cytosolic fraction.

### Immunofluorescence Staining

HCFs were plated on 6-well culture plates with coverslips. Cells were shifted to a serum-free DMEM/F-12 for 24 h and treated with 15 μM S1P. The cells were washed twice with ice-cold PBS, then fixed with 4% (w/v) paraformaldehyde in PBS for 30 min and permeabilized with 0.3% Triton X-100 in PBS for 15 min. Next, the staining procedures were performed by incubating with 10% normal goat serum in PBS for 30 min, followed by incubating with a primary anti-p65 NF-κB polyclonal antibody (1:200 dilution) in PBS with 1% BSA for 1 h. After washing thrice with PBS, the cells were incubated with fluorescein isothiocyanate (FITC)-conjugated goat anti-rabbit antibody (1:200 dilution) in PBS with 1% BSA for 1 h. Finally, after washing thrice with PBS, the cells were mounted with aqueous mounting medium. The images were taken under a fluorescence microscope (ZEISS, Axiovert 200M).

### Chromatin Immunoprecipitation Assay

To detect the *in vivo* association of nuclear proteins with human COX-2 promoter, chromatin immunoprecipitation (ChIP) analysis was conducted as previously described (Lin et al., 2013). Briefly, HCFs were cross-linked with 1% formaldehyde for 10 min at 37°C. According to the manufacturer’s recommendations, soluble chromatin was prepared using a ChIP assay kit (Upstate, Essex County, NY, United States) after washed thrice with ice-cold PBS containing 1 mM phenylmethylsulfonyl fluoride (PMSF) and 1% aprotinin. Then, the soluble chromatin was immunoprecipitated with anti-p65 NF-κB antibody or without (control) and normal goat immunoglobulin G (IgG). The precipitates were heated overnight at 65°C to release DNA from crosslinking protein after washes and elution. Phenol-chloroform extraction and ethanol precipitation were used to purify DNA fragments. Then, the primers specific for the region containing the NF-κB binding site present in the COX-2 promoter region were used to amplify the purified DNA by PCR. Two percent agarose in 1× TAE gel containing ethidium bromide was used to analyze the amplified PCR fragments and the size (279 bp) was compared to a molecular weight marker.

### Measurement of Prostaglandin E_2_ Release

The cells in 12-well plates were grown to confluence. Then, cells were incubated with serum-free DMEM/F-12 medium for 24 h. After that, the growth-rested cells were treated with S1P as indicated time intervals. The conditioned medium was collected to analyze PGE_2_ levels by using an EIA kit as specified by the manufacturer (Cayman Chemicals).

### Statistical Analysis of Data

Statistical analysis was performed by using GraphPad Prizm Program 6.0 software (GraphPad, San Diego, CA, United States). We used one-way ANOVA followed by Dunnett’s post hoc test when comparing more than two groups of data and one-way ANOVA, nonparametric Kruskal–Wallis test, followed by Dunnett’s post hoc test when comparing multiple independent groups. *p* values of 0.05 were considered to be statistically significant. Post hoc tests were run only if F achieved *p* < 0.05 and there was no significant in homogeneity of variance. All the data were expressed as the mean ± SEM, at least three individual experiments (n = number of independent cell culture preparations). The n values are provided in the figure legends. Error bars were omitted when they fell within the dimensions of the symbols.

## Results

### Sphingosine 1-Phosphate Induces Cyclooxygenase-2 Expression and Prostaglandin E_2_ Synthesis

**FIGURE 1:**
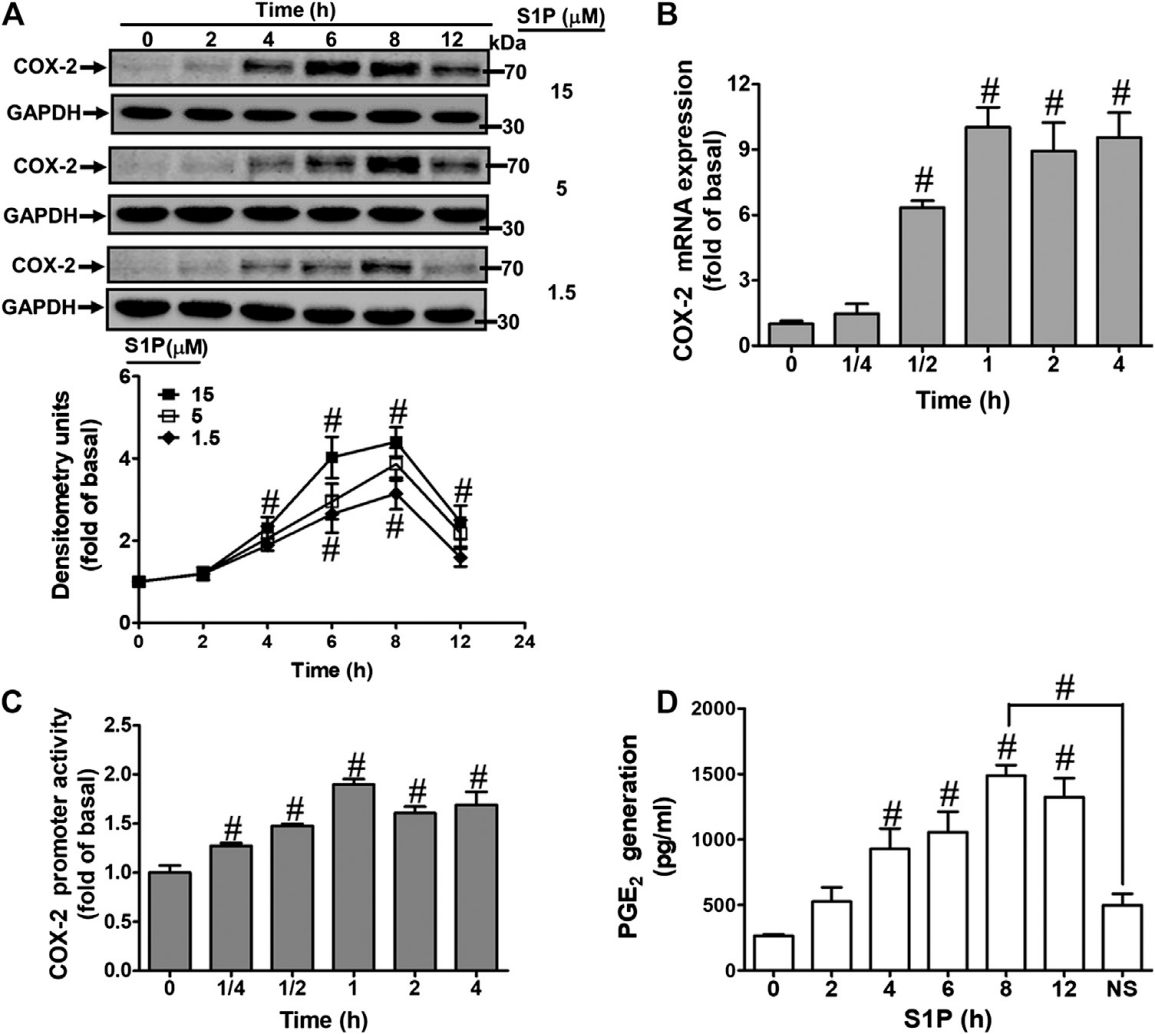
S1P induced COX-2 expression and PGE_2_ generation in HCFs. **(A)** Cells were incubated with various concentrations of S1P for the indicated time intervals. **(B)** Cells were incubated with 15 μM S1P for the indicated time intervals. COX-2 mRNA was analyzed by real time-PCR. **(C)** Cells were transfected with a COX-2 promoter-luciferase reporter gene and then incubated with 15 μM S1P for the indicated time intervals. The promoter reporter assay was determined. **(D)** Various concentrations of S1P were used to treat the cells for the indicated time intervals. In addition, cells were pretreated with NS398 (10 μM) for 1 h and then incubated with 15 μM S1P for 8 h. The media were collected to determine PGE_2_ levels by EIA. Data are expressed as mean ± SEM of three individual experiments (n = 3). ^#^ *p* < 0.05, as compared with the cells treated with vehicle (DMSO) alone.

To evaluate the effect of S1P on COX-2 expression, HCFs were challenged with S1P as the indicated concentrations and time intervals. As shown in Figure 1A, S1P time- and concentration-dependently induced COX-2 protein expression. A significant increase response was observed within 4 h and a maximal response at 8 h. To further examine whether S1P-induced COX-2 expression is mediated through COX-2 mRNA expression, the real time-PCR was performed. The data showed that S1P-induced COX-2 mRNA expression was significantly increased within 30 min, reached a maximal response within 1 h, and sustained for 4 h during the period of observation (Figure 1B). Moreover, we also confirmed that S1P induced COX-2 expression *via* turning on COX-2 gene expression determined by a COX-2-promoter luciferase report assay. As shown in Figure 1C, S1P stimulated COX-2 promoter activity in a time-dependent manner and reached a significant increase within 0.5–1 h. Here we further determined whether S1P-enhanced the amount of COX-2 protein was accompanied by a corresponding generation of PGE_2_, the cultured media were collected to measure PGE_2_ levels using an ELISA kit. The data showed that S1P enhanced PGE_2_ generation in a time-dependent manner (Figure 1D). There was a significant increase within 4 h and reached a maximum at 8 h, which was attenuated by pretreatment with a COX-2 activity inhibitor NS-398 (10 μM), indicating that S1P induced a COX-2-dependent PGE_2_ generation in HCFs. These results suggested that S1P induces COX-2 expression via up-regulation of COX-2 gene, which enhances PGE_2_ production in HCFs.

### Sphingosine 1-Phosphate-Induced Cyclooxygenase-2 Expression Is Mediated Through Sphingosine 1-PhosphateR1/3

**FIGURE 2:**
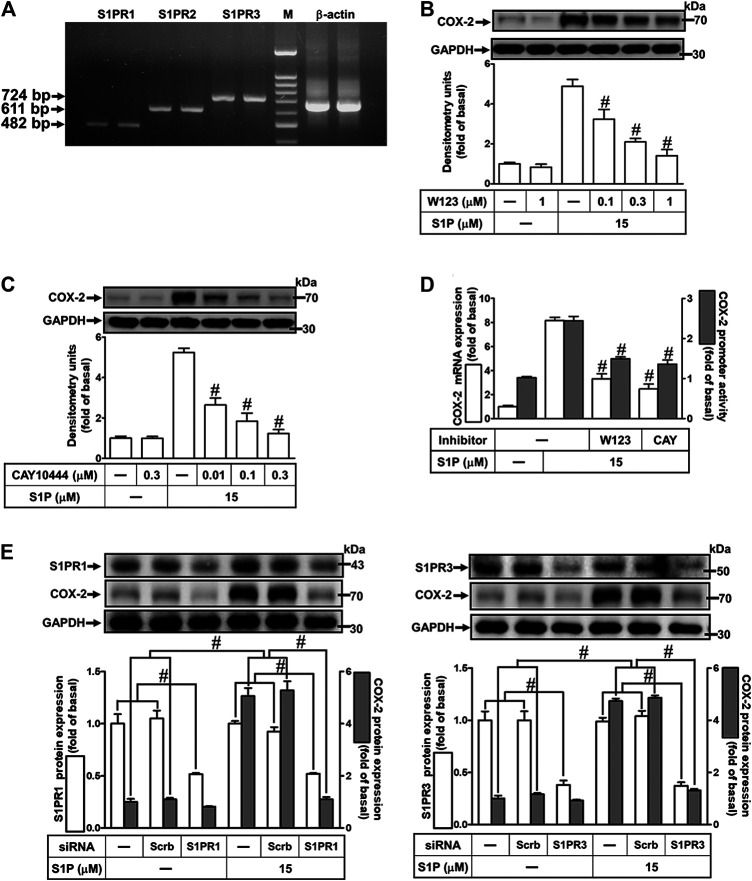
Involvement of S1PR1 and S1PR3 in S1P-induced COX-2 expression. **(A)** RT-PCR analysis of S1PRs expression in HCFs. Lane 1–2: S1PR1; Lane 3–4: S1P2; Lane 5–6: S1P3; Lane 7: marker; Lane 8–9: *β*-actin. **(B,C)** Cells were pretreated with W123 or CAY10444 for 1 h and then incubated with 15 μM S1P for 8 h. **(D)** Cells were transfected without or with COX-2 promoter-luciferase reporter gene, pretreated without or with W123 (1 μM) or CAY10444 (0.3 μM) for 1 h and then incubated with 15 μM S1P for 4 h (mRNA) or 1 h (promoter). **(E)** Cells were transfected with siRNA for scrambled, S1PR1, or S1PR3 for 24 h and then exposed to 15 μM S1P for 8 h. **(B,C,E)** The levels of COX-2, S1PR1, S1PR3, and GAPDH protein were analyzed by Western blot. **(D)** The levels of COX-2 mRNA and promoter activity were analyzed by real time-PCR (open bar) and promoter assay (gray bar). Data are expressed as mean ± SEM of three individual experiments (n = 3). ^#^ *p* < 0.05, as compared with the cells treated with S1P alone.

Several biological responses induced by S1P are mediated through its receptors, including S1PR1, S1PR2, and S1PR3 (Means and Brown, 2009). Hence, the expression of S1P receptor subtypes on HCFs was determined by RT-PCR analysis. As shown in Figure 2A, all of these S1PR subtypes were expressed on HCFs. Furthermore, to investigate whether these receptors are involved in S1P-induced COX-2 expression and PGE_2_ production, selective antagonists of S1P receptor subtypes were used for this purpose. As shown in Figures 2B,C, pretreatment of HCFs with either W123 (S1PR1 antagonist) or CAY10444 (S1PR3 antagonist), but not JTE-013 (S1PR2 antagonist, data not shown), concentration-dependently attenuated the S1P-induced COX-2 protein expression. Consistently, S1P-induced COX-2 mRNA expression and transcription activity were also attenuated by W123 or CAY10444 (Figure 2D), suggesting that S1PR_1_ and S1PR_3_ are involved in S1P-induced COX-2 expression. Moreover, to confirm the involvement of S1PR1 and S1PR3 in S1P-induced responses, as shown in Figure 2E, transfection with S1PR1 or S1PR3 knocked down S1PR1 or S1PR3 protein expression, respectively, and both attenuated S1P-induced COX-2 expression. These results suggested that S1PR1 and S1PR3 are involved in S1P-induced COX-2 expression in HCFs.

### Involvement of G_q_ and G_i_ Protein-Coupled S1PRs in Sphingosine 1-Phosphate-Induced Cyclooxygenase-2 Expression

**FIGURE 3:**
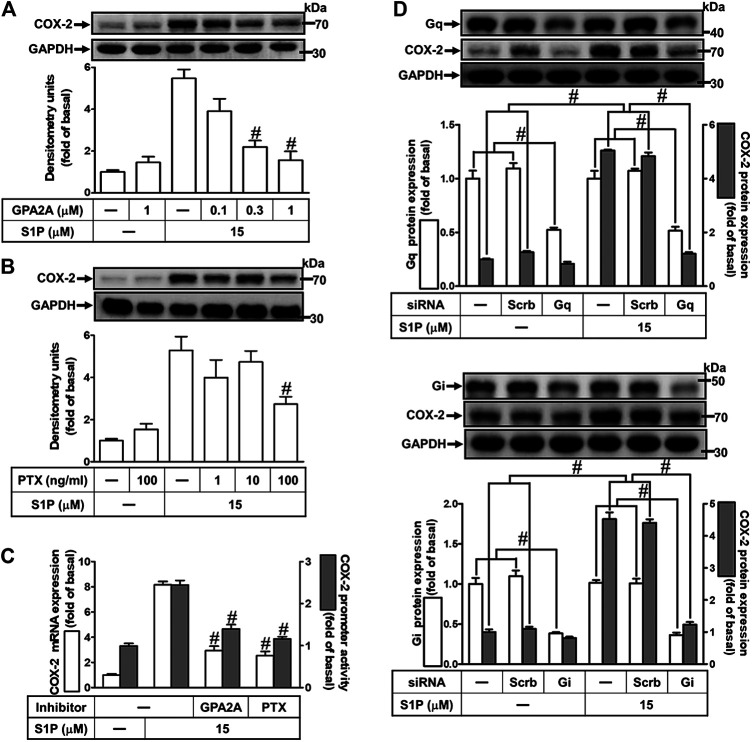
S1P induces COX-2 expression via a G_q_- or PTX-sensitive G_i_ protein-coupled S1PR1/3. **(A,B)** Cells were pretreated with GPA2A (G_q_ antagonist) for 1 h or pertussis toxin (PTX, G_i_ protein inactivator) for 24 h and then incubated with 15 μM S1P for 8 h. **(C)** Cells were transfected without or with COX-2 promoter-luciferase reporter gene, pretreated with GPA2A (1 μM) or PTX (100 ng/ml) for 1 h, and then incubated with 15 μM S1P for 4 h (mRNA) or 1 h (promoter). The COX-2 mRNA and promoter activity were analyzed by real time-PCR (open bar) and promoter assay (gray bar). **(D)** Cells were transfected with siRNA for scrambled, G_q_α, or G_i_α for 24 h and then exposed to 15 μM S1P for 8 h **(A,B,D)** The levels of COX-2, GAPDH, G_q_α, and G_i_α proteins were analyzed by Western blot. Data are expressed as mean ± SEM of three individual experiments (n = 3). ^#^ *p* < 0.05, as compared with the cells treated with S1P alone.

Extracellular S1P interacts with a family of specific G protein-coupled receptors (GPCRs) to evoke multiple cellular responses. First, to determine whether G_q_ protein is involved in S1P-induced COX-2 expression, a G_q_ protein antagonist (GPA2A) was used. As shown in Figure 3A, pretreatment with GPA2A concentration-dependently attenuated S1P-induced COX-2 expression. Next, we also demonstrated the role of G_i_ in the response, a pertussis toxin (PTX) was used. As shown in Figure 3B, pretreatment with PTX for overnight attenuated the S1P-induced COX-2 protein. Similarly, pretreatment with either GPA2A or PTX inhibited S1P-induced COX-2 mRNA expression and transcription activity (Figure 3C). To further ensure the involvement of G_q_ and G_i_ in S1P-induced COX-2 expression, HCFs were transfected with G_q_ or G_i_ siRNA. As shown in Figure 3D, transfection with G_q_ or G_i_ siRNA knocked down the level of G_q_ or G_i_ protein expression, respectively, and attenuated S1P-induced COX-2 expression. These results indicated that S1P induces COX-2 expression *via* S1PR1 and S1PR3 coupling to either G_q_ protein or PTX-sensitive G_i_ protein in HCFs.

### PKCα Is Involved in Sphingosine 1-Phosphate-Induced Cyclooxygenase-2 Expression

**FIGURE 4:**
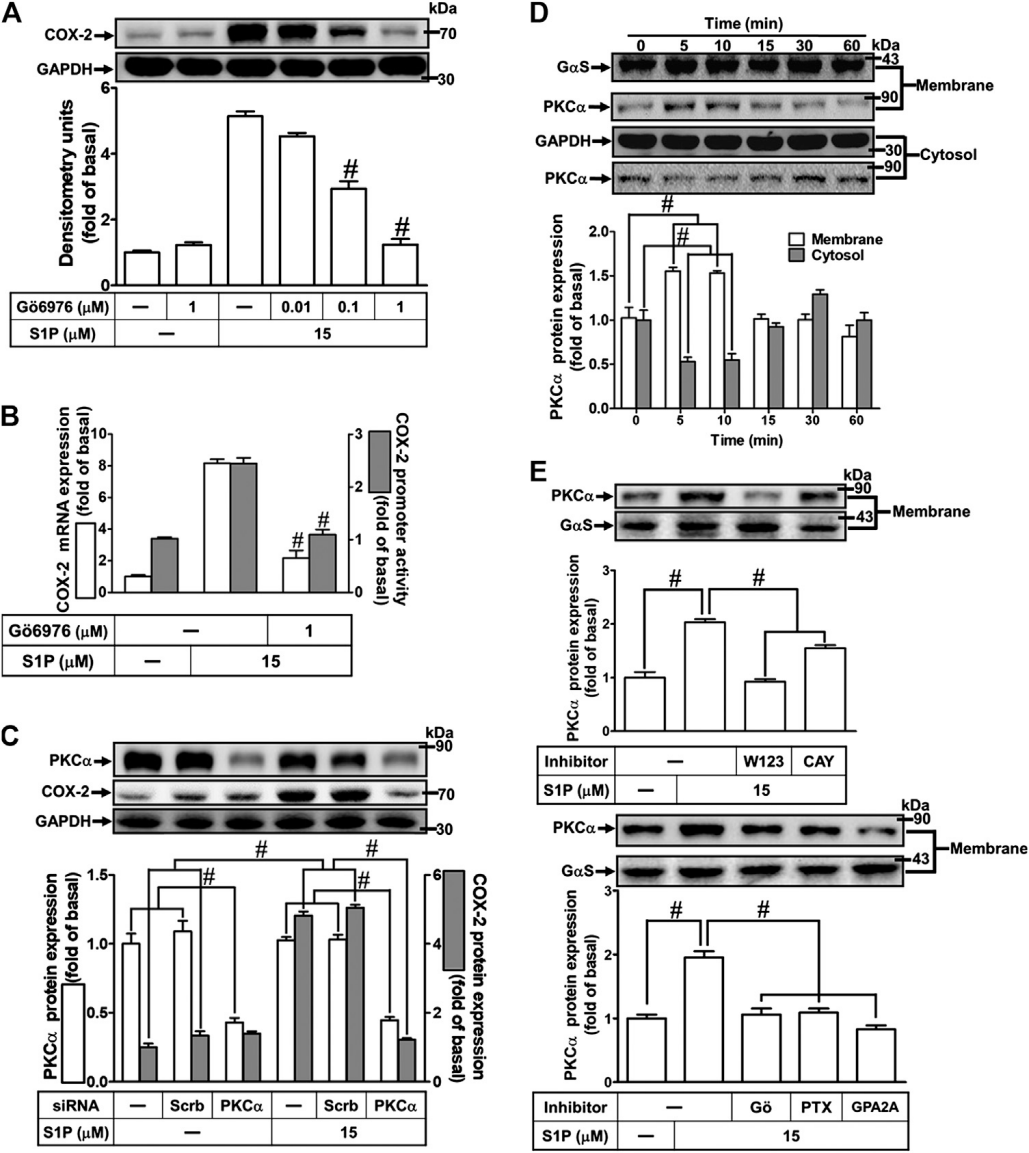
PKCα mediates S1P-induced COX-2 expression. **(A)** Cells were pretreated with Gö6976 for 1 h and then incubated with 15 μM S1P for 8 h. **(B)** Cells were transfected without or with COX-2 promoter-luciferase reporter gene, pretreated without or with Gö6976 (1 μM) for 1 h, and then incubated with 15 μM S1P for 4 h (mRNA) or 1 h (promoter). Western blot, real time-PCR, and promoter assay were performed to determine the levels of COX-2 protein, mRNA expression, and promoter activity, respectively. **(C)** Prior to exposure with S1P for 8 h, cells were transfected with siRNA of scrambled or PKCα. **(D)** Cells were incubated with S1P (15 μM) for the indicated time intervals. **(E)** Cells were incubated with S1P (15 μM) for 5 min in the absence or presence of Gö6976 (1 μM), W123 (10 μM), CAY (10 μM), GPA2A (10 μM), or PTX (100 ng/ml) for 1 h. The membrane and cytosol fractions were prepared and analyzed by Western blot. Data are expressed as mean ± SEM of three individual experiments (n = 3). ^#^ *p* < 0.05, as compared with the cells treated with S1P alone.

The classic PKC isoforms such as PKCα have been demonstrated to participate in the regulation of several proteins expression in various cell types (Lin et al., 2012). Hence, to investigate whether PKCs, PKCα especially, are involved in S1P-induced responses, a selective PKCα inhibitor Gö6976 was used. As shown in Figure 4A, pretreatment with Gö6976 concentration-dependently inhibited S1P-induced COX-2 protein expression. Moreover, pretreatment with Gö6976 also attenuated S1P-induced COX-2 mRNA expression and transcription activity (Figure 4B), suggesting that PKCα plays a key role in S1P-induced COX-2 gene expression in HCFs. To further ensure the role of PKCα in COX-2 expression, as shown in Figure 4C, PKCα protein expression was significantly knocked down by transfection with PKCα siRNA, which also attenuated COX-2 expression induced by S1P. Furthermore, S1P-induced PKCα translocation from the cytosol to the membrane responses was further confirmed in our study. The data showed that S1P time-dependently stimulated PKCα translocation and reached a maximal response within 5 min (Figure 4D). Moreover, we demonstrated whether S1P-stimulated PKCα translocation is mediated through a G_q_/G_i_ protein-coupled S1PR1/3 signaling pathway. As shown in Figure 4E, S1P-stimulated PKCα translocation was attenuated by pretreatment with W123 (1 μM), CAY (0.3 μM), Gö6976 (1 μM), GPA2A (1 μM), or PTX (100 ng/ml). These results suggested that S1P induces COX-2 expression via the G_q_/G_i_ protein-coupled S1PR_1/3_-mediated activation of PKCα in HCFs.

### Role of p42/p44 Mitogen-Activated Protein Kinases in Sphingosine 1-Phosphate-Induced Cyclooxygenase-2 Expression

**FIGURE 5:**
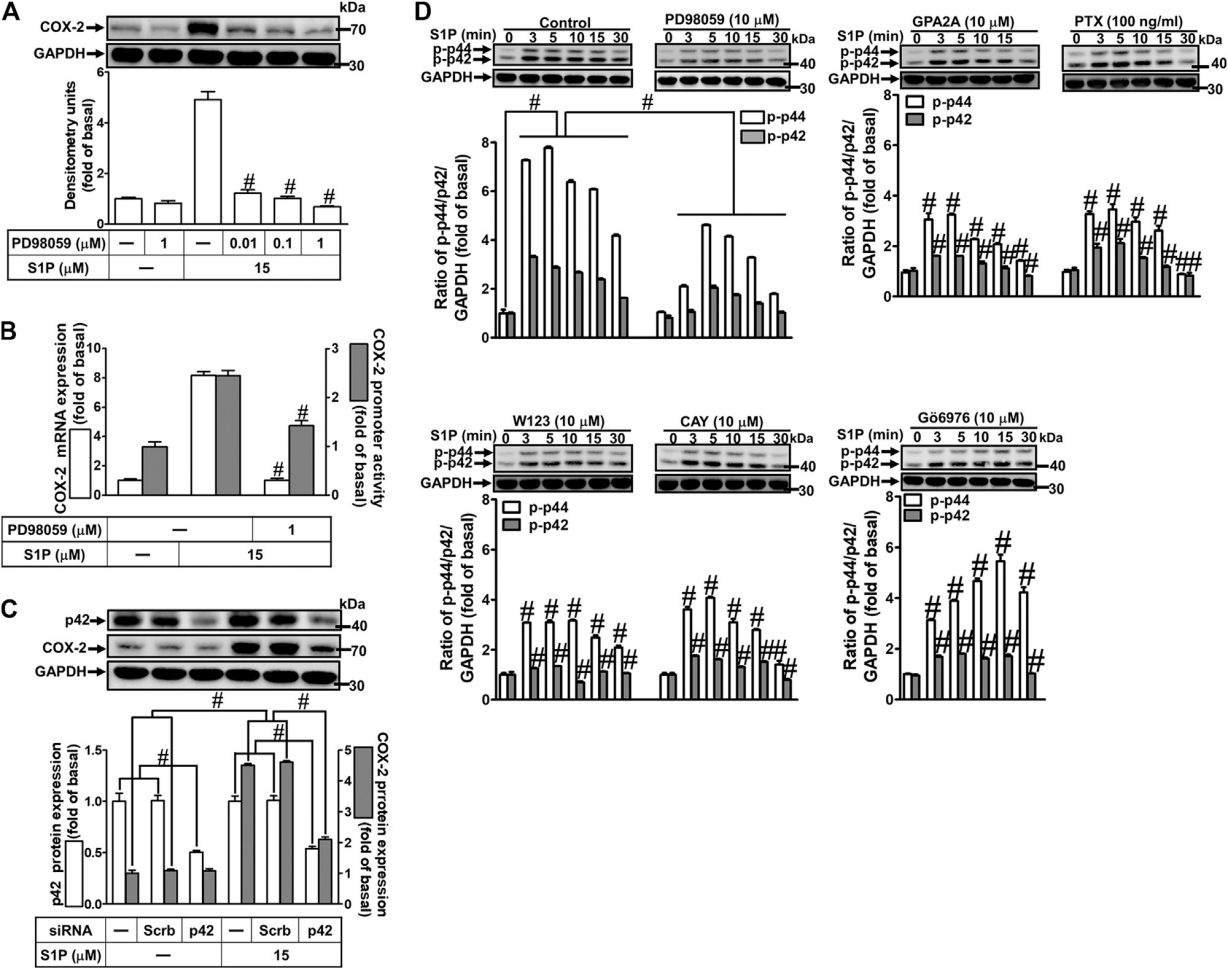
S1P-induced COX-2 expression is mediated through p42/p44 MAPK phosphorylation. **(A)** Cells were pretreated with PD98059 for 1 h and then incubated with 15 μM S1P for 8 h. **(B)** Cells were transfected without or with COX-2 promoter-luciferase reporter gene, pretreated without or with PD98059 (10 μM) for 1 h, and then incubated with 15 μM S1P for 4 h (mRNA) or 1 h (promoter). The levels of COX-2 protein, mRNA expression, and promoter activity were determined by Western blot, real time-PCR, and promoter assay, respectively. **(C)** Cells were transfected with siRNA of scrambled or p42 MAPK and then exposed to S1P for 8 h. **(D)** Cells were incubated with S1P (15 μM) for the indicated time intervals in the absence or presence of PD98059 (10 μM), W123 (10 μM), CAY (10 μM), GPA2A (10 μM), PTX (100 ng/ml), or Gö6976 (10 μM). The cell lysates were collected and analyzed by Western blot. The fold of basal was defined as normalization of the data to the respective “0,” and then compared the data of corresponding time points of control vs inhibitor with a statistic method, as described in the section of *Methods*. Data are expressed as mean ± SEM of three individual experiments (n = 3). ^#^ *p* < 0.05, as compared with the cells treated with S1P alone.

Activation of MAPKs by S1P could modulate cellular functions of vascular smooth muscle cells (Pyne and Pyne, 2000). To investigate the role of p42/p44 MAPK in S1P-induced COX-2 expression in HCFs, a MEK1/2 (an upstream component of p42/p44 MAPK) inhibitor PD98059 was used. As shown in Figure 5A, pretreatment with PD98059 dose-dependently attenuated S1P-induced COX-2 expression. Moreover, pretreatment with PD98059 attenuated S1P-induced COX-2 mRNA expression and transcription activity (Figure 5B), suggesting that p42/p44 MAPK is involved in S1P-induced COX-2 gene expression in HCFs. We further ensured the role of p42/p44 MAPK in COX-2 expression, as shown in Figure 5C, cells were transfected with p42 MAPK siRNA significantly knocked down p42 protein level and attenuated S1P-induced COX-2 expression. Furthermore, to determine whether the activation of p42/p44 MAPK was required for S1P-induced responses, as shown in Figure 5D, S1P time-dependently stimulated p42/p44 MAPK phosphorylation and reached a maximal response within 3 min, which was markedly attenuated by pretreatment with PD98059 during the period of observation. The data indicated that p42/p44 MAPK contributes to S1P-induced COX-2 expression in HCFs. In addition, pretreatment with W123 (10 μM), CAY (10 μM), GPA2A (10 μM), PTX (100 ng/ml), or Gö6976 (10 μM) also attenuated S1P-stimulated p42/p44 MAPK phosphorylation, suggesting that S1P-stimulated p42/p44 MAPK phosphorylation is mediated through G (G_q_/G_i_) protein-coupled S1PR1/3-mediated PKCα pathway leading to COX-2 expression in HCFs.

### Role of p38 Mitogen-Activated Protein Kinases in Sphingosine 1-Phosphate-Induced Cyclooxygenase-2 Expression

**FIGURE 6:**
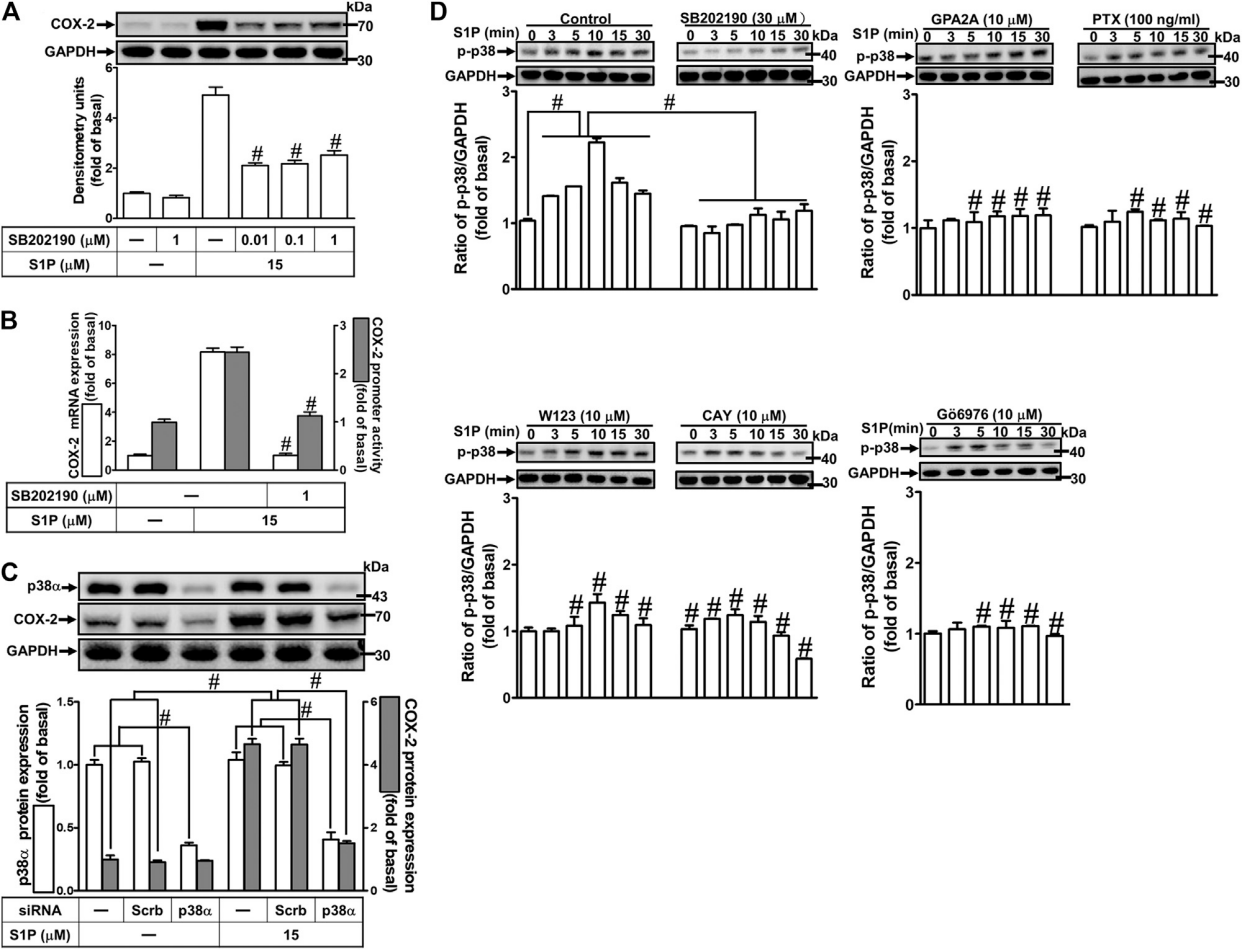
S1P-induced COX-2 expression is mediated through p38 MAPK phosphorylation. **(A)** Cells pretreated with SB202190 for 1 h were followed by incubation with 15 μM S1P for 8 h. **(B)** Cells were transfected without or with COX-2 promoter-luciferase reporter gene, pretreated without or with SB202190 (30 μM) for 1 h, and then incubated with 15 μM S1P for 4 h (mRNA) or 1 h (promoter). The levels of COX-2 protein, mRNA expression, and promoter activity were individually examined by Western blot, real time-PCR, and promoter assay. **(C)** Cells transfected with p38α MAPK siRNA were incubated with S1P for 8 h. (**D**) Cells were incubated with S1P (15 μM) for the indicated time intervals in the absence or presence of SB202190 (30 μM), W123 (10 μM), CAY (10 μM), GPA2A (10 μM), or Gö6976 (10 μM) for 1 h and PTX (100 ng/ml) for 24 h. The cell lysates were collected and analyzed by Western blot. The fold of basal was defined as normalization of the data to the respective “0,” and then compared the data of corresponding time points of control vs inhibitor with a statistic method, as described in the section of *Methods*. Data are expressed as mean ± SEM of three individual experiments (n = 3). ^#^ *p* < 0.05, as compared with the cells treated with S1P alone.

To investigate the effect of p38 MAPK on S1P-induced COX-2 expression in HCFs, a p38 MAPK inhibitor SB202190 was used. As shown in Figure 6A, pretreatment with SB202190 concentration-dependently attenuated S1P-induced COX-2 expression. Moreover, pretreatment with SB202190 attenuated S1P-induced COX-2 mRNA expression and transcription activity (Figure 6B), suggesting that p38 MAPK plays a crucial role in S1P-induced COX-2 gene expression in HCFs. To further ensure the role of p38 MAPK in COX-2 expression, as shown in Figure 6C, cells were transfected with p38 MAPK siRNA significantly knocked down p38 MAPK protein level and attenuated the S1P-induced COX-2 expression. Furthermore, to determine whether the activation of p38 MAPK was required for S1P-induced responses, as shown in Figure 6D, S1P stimulated p38 MAPK phosphorylation in a time-dependent manner with a maximal response within 10 min, which was markedly attenuated by pretreatment with SB202190 (30 μM) during the period of observation, suggesting that p38 MAPK is involved in S1P-induced COX-2 expression. In addition, pretreatment with W123 (10 μM), CAY (10 μM), GPA2A (10 μM), PTX (100 ng/ml), or Gö6976 (10 μM) attenuated S1P-stimulated p38 MAPK phosphorylation (Figure 6D), suggesting that S1P-stimulated p38 MAPK phosphorylation is involved in S1P-induced responses *via* the G (G_q_/G_i_) protein-coupled S1PR1/3-mediated PKCα pathway leading to COX-2 expression in HCFs.

### Role of c-Jun N-Terminal Kinase in Sphingosine 1-Phosphate-Induced Cyclooxygenase-2 Expression

**FIGURE 7:**
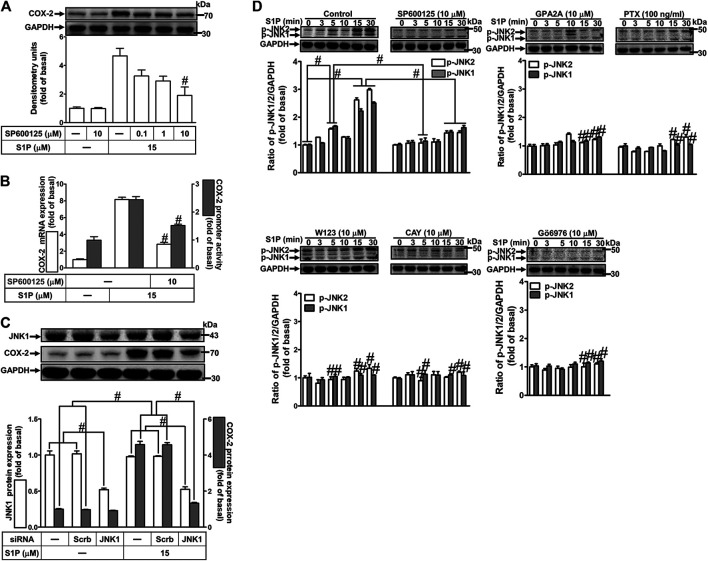
JNK1/2 phosphorylation is involved in S1P-induced COX-2 expression. **(A)** After pretreatment with SP600125 for 1 h, cells were challenged with 15 μM S1P for 8 h. **(B)** Cells were transfected without or with COX-2 promoter-luciferase reporter gene, pretreated without or with SP600125 (10 μM) for 1 h, and then incubated with 15 μM S1P for 4 h (mRNA) or 1 h (promoter). The levels of COX-2 protein, mRNA expression, and promoter activity were determined by Western blot, real time-PCR, and promoter assay, respectively. **(C)** Cells were transfected with siRNA of JNK1 and then exposed to S1P for 8 h. **(D)** Cells were incubated with S1P (15 μM) for the indicated time intervals in the absence or presence of SP600125 (10 μM), W123 (10 μM), CAY (10 μM), GPA2A (10 μM), PTX (100 ng/ml), or Gö6976 (10 μM) for 1 h. The cell lysates were collected and analyzed by Western blot. The fold of basal was defined as normalization of the data to the respective “0,” and then compared the data of corresponding time points of control vs inhibitor with a statistic method, as described in the section of *Methods*. Data are expressed as mean ± SEM of three individual experiments (n = 3). ^#^ *p* < 0.05, as compared with the cells treated with S1P alone.

To determine the role of JNK1/2 in S1P-induced COX-2 expression, a JNK1/2 inhibitor SP600125 was used. As shown in Figure 7A, pretreatment with SP600125 concentration-dependently attenuated S1P-induced COX-2 expression. Moreover, pretreatment with SP600125 attenuated S1P-induced COX-2 mRNA expression and transcription activity (Figure 7B), suggesting that JNK1/2 is crucial for S1P-induced COX-2 gene expression in HCFs. To further ensure the role of JNK1/2 in COX-2 expression, as shown in Figure 7C, cells were transfected with JNK1 siRNA markedly knocked down JNK1 protein level and attenuated S1P-induced COX-2 expression. Furthermore, we determine whether S1P-stimulated JNK activation was required for these responses, the activation of JNK1/2 was determined by Western blot using an anti-phospho-JNK1/2 antibody. As shown in Figure 7D, S1P time-dependently stimulated JNK1/2 phosphorylation with a maximal response within 15–30 min, which was markedly attenuated by pretreatment with SP600125 during the period of observation, suggesting that JNK1/2 phosphorylation contributes to S1P-induced COX-2 expression. In addition, pretreatment with W123 (10 μM), CAY (10 μM), GPA2A (10 μM), PTX (100 ng/ml), or Gö6976 (10 μM) attenuated S1P-stimulated JNK1/2 phosphorylation (Figure 7D). These results suggested that S1P-induced JNK1/2-mediated COX-2 expression is mediated through the G (G_q_/G_i_) protein-coupled S1PR1/3-mediated PKCα pathway in HCFs.

### NF-κB Is Required for Sphingosine 1-Phosphate-Induced Cyclooxygenase-2 Expression

**FIGURE 8:**
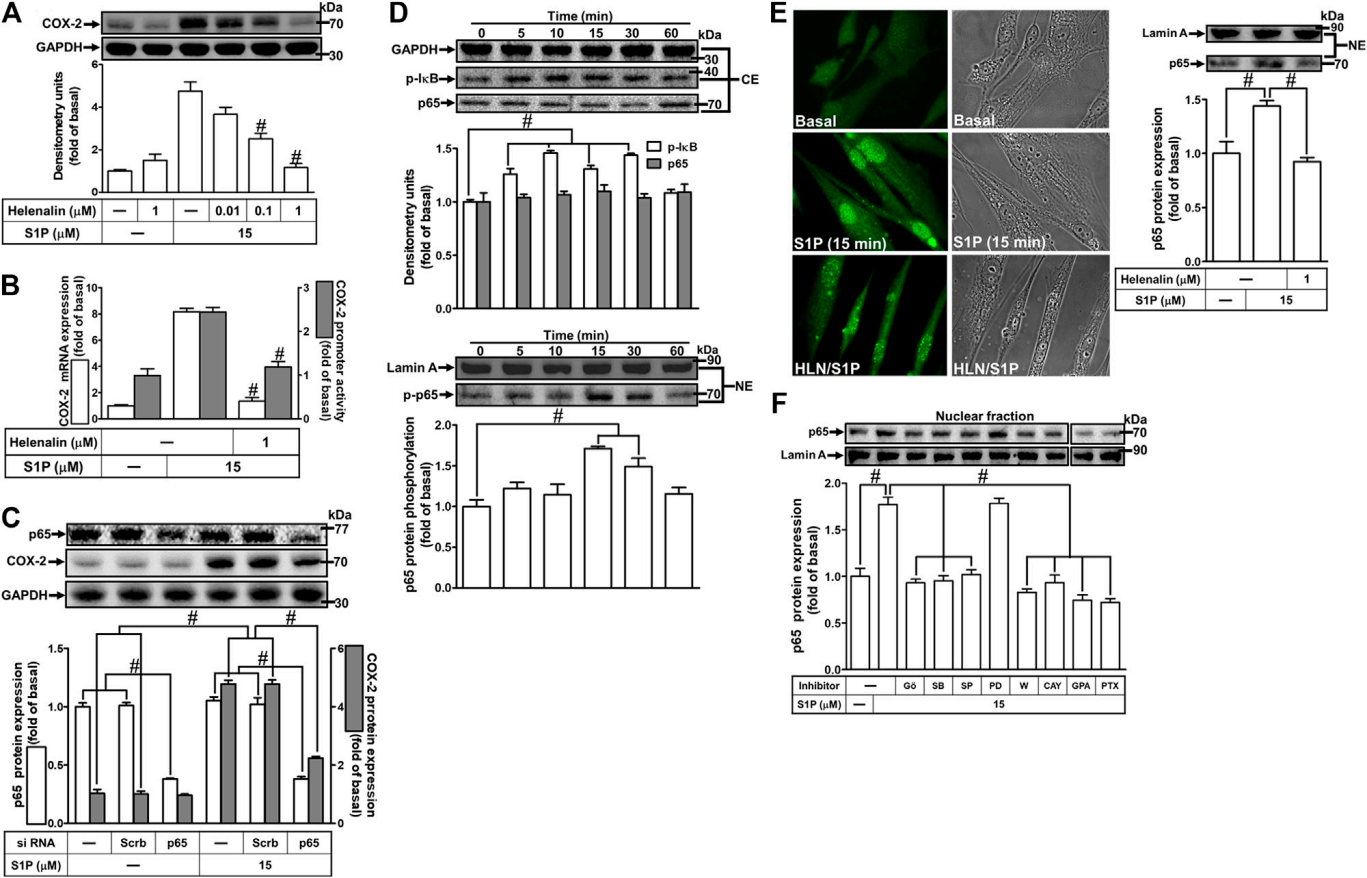
NF-κB is essential for S1P-induced COX-2 expression. **(A)** Cells were pretreated with helenalin for 1 h and then incubated with 15 μM S1P for 8 h. **(B)** Cells were transfected without or with COX-2 promoter-luciferase reporter gene, pretreated without or with helenalin (1 μM) for 1 h, and then incubated with 15 μM S1P for 4 h (mRNA) or 1 h (promoter). The levels of COX-2 protein, mRNA expression, and promoter activity were determined by Western blot, real time-PCR, and promoter assay, respectively. **(C)** Cells were transfected with siRNA of p65 and then exposed to S1P for 8 h. **(D)** Cells were incubated with S1P (15 μM) for the indicated time intervals. **(E)** Cells were treated with S1P (15 μM) for 15 min in the absence or presence of helenalin (1 μM). **(D,E)** The nuclear and cytosol fractions were prepared and analyzed by Western blot. The p65 NF-κB translocation by S1P was also determined by immunofluorescent staining as described in *Materials and Methods*. **(F)** To determine which S1PR subtypes, Gi or Gq protein, and MAPKs involved in S1P-stimulated the nuclear localization of p65 NF-κB, cells were incubated with S1P (15 μM) for 15 min in the absence or presence of Gö6976 (Gö, 10 μM), SB202190 (SB, 30 μM), SP600125 (SP, 10 μM), PD98059 (PD, 10 μM), W123 (W, 10 μM), CAY (10 μM), GPA2A (GPA, 10 μM), or PTX (100 ng/ml), and the nuclear fraction was analyzed by Western blot. To fit the construct of data layout, the data were rearranged from the same gel with the exception of non-related inhibitors and disclosed by the insertion of white spaces rearranged from the original capture. Data are expressed as mean ± SEM of three individual experiments (n = 3). ^#^ *p* < 0.05, as compared with the cells treated with S1P alone.

S1P could activate NF-κB signaling to regulate cellular functions in various cell types (Takuwa et al., 2012). To prove activation of NF-κB is required for COX-2 expression induced by S1P, pretreatment with a selective NF-κB inhibitor helenalin, which blocks activation of NF-κB signaling, attenuated S1P-induced COX-2 protein (Figure 8A), mRNA expression and transcription activity (Figure 8B) in HCFs. To further verify that NF-κB is essential for S1P-induced COX-2 expression, as shown in Figure 8C, transfection with p65 (an NF-κB subunit) siRNA significantly reduced the p65 protein expression and the S1P-induced COX-2 expression. Moreover, to determine whether the p65 NF-κB translocation is involved in S1P-induced responses, as shown in Figure 8D, S1P time-dependently stimulated either phosphorylation of I-κB or translocation of p65 NF-κB from the cytosol into the nucleus determined by Western blot. A maximal response was observed within 10–30 min. The p65 translocation by S1P was inhibited by pretreatment with helenalin (Figure 8E, left panel). Moreover, the p65 NF-κB translocation was also confirmed by an immunofluorescence staining. The data demonstrated that S1P stimulated the p65 nuclear translocation at 15 min (Figure 8E, right panel), which was attenuated by pretreatment with helenalin. We also demonstrated that S1P-stimulated translocation of p65 NF-κB was attenuated by pretreatment with W123, CAY, GPA2A, PTX, Gö6976, PD98059, SB202190, or SP600125 (Figure 8F). These results suggested that S1P-stimulated NF-κB translocation is mediated through S1PR1/3, G_q_/G_i_, PKCα, p38 MAPK, and JNK1/2, but not p42/p44 MAPK, leading to COX-2 induction in HCFs.

### Involvement of NF-κB in Cyclooxygenase-2 Gene Promoter Activity Induced by Sphingosine 1-Phosphate

**FIGURE 9:**
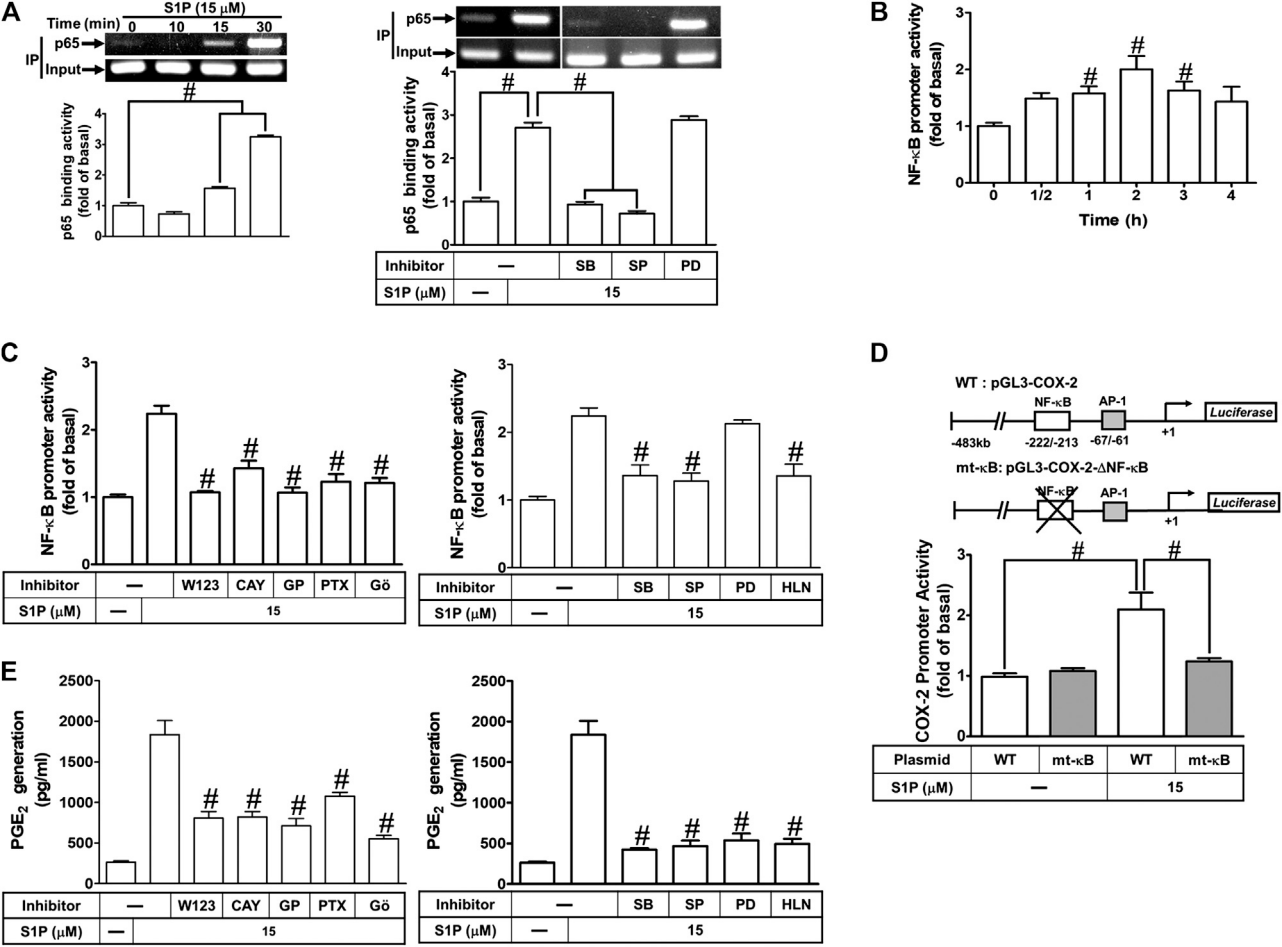
COX-2 promoter activity is stimulated by S1P through an NF-κB-dependent pathway. **(A)** Cells were incubated with S1P for the indicated time intervals (upper panel). Cells were pretreated with PD98059 (10 μM), SB202190 (30 μM), SP600125 (10 μM) for 1 h and then incubated with S1P for 30 min. The binding activity of NF-κB p65 and promoter was analyzed by ChIP assay (n = 3), as described in *Methods*. Left panel, to fit the construct of data layout, the data were rearranged from the same gel with the exception of non-related inhibitors and disclosed by the insertion of white spaces rearranged from the original capture. **(B,C)** Cells were transfected with an NF-κB-luciferase reporter gene, pretreated with W123 (10 μM), CAY (10 μM), GPA2A (10 μM), PTX (100 ng/ml), Gö6976 (10 μM), PD98059 (10 μM), SB202190 (30 μM), SP600125 (10 μM), or helenalin (1 μM) for 1 h and then incubated with S1P for 2 h. **(D)** The schematic picture represented two different 5′-promoter regions of the mouse COX-2 promoter constructs, both wild-type (WT) and mt-κB modified by single-point mutation of the κB binding site fused to the pGL-luciferase reporter gene. “↲ ” indicated the translational start site (+1) of the luciferase reporter gene. WT COX-2 promoter reporter gene (WT-COX-2) or NF-κB mutated COX-2 promoter reporter gene (mt-κB-COX-2) were transfected into cells, which then were incubated with or without S1P for 1 h. The promoter reporter activity was determined. **(E)** Cells were pretreated with W123, CAY, GPA2A, PTX, Gö6976, PD98059, SB202190, SP600125, or helenalin for 1 h and then incubated with S1P for 8 h. The PGE_2_ levels were analyzed by EIA. Data are expressed as mean ± SEM of three individual experiments (n = 3). ^#^ *p* < 0.05, as compared with the cells treated with S1P alone.

We have found that S1P stimulates translocation of p65 NF-κB leading to COX-2 expression (Figure 8). Next, we examined whether translocated p65 NF-κB can bind to the COX-2 promoter region, a ChIP-PCR analysis was performed. As shown in Figure 9A (upper panel), S1P-stimulated p65 NF-κB binding activity in a time-dependent manner which was attenuated by pretreatment with SB202190 or SP600125, but not PD98059 (Figure 9A, lower panel). These results suggested that S1P-stimulated p65 NF-κB binding activity is mediated through p38 MAPK- and JNK1/2-dependent pathways. We further examined whether activation of NF-κB was essential for S1P-induced COX-2 gene up-regulation. We used a promoter (containing NF-κB binding sites)-luciferase activity assay to evaluate the transcription activity of NF-κB. The data showed that S1P enhanced NF-κB transcription activity in a time-dependent manner with a maximal response within 2 h (Figure 9B), which was significantly inhibited by pretreatment with W123, CAY, GPA2A, PTX, Gö6976, SB202190, SP600125, or helenalin, but not PD98059 (Figure 9C). These results demonstrated that S1P enhances the NF-κB transcription activity through an S1PR1/3-dependent activation of PKCα/MAPKs (i.e., p38 MAPK and JNK1/2) cascades. In addition, to further confirm that NF-κB through binding to its regulatory element on the COX-2 promoter region participates in the S1P-induced COX-2 promoter activity, we constructed both the wild-type (WT) and mt-κB COX-2 promoters mutated by a single-point mutation of the NF-κB binding site (Figure 9D, upper panel). Transfection of HCFs with mt-κB-COX-2 plasmid significantly inhibited the S1P-stimulated COX-2 promoter activity (Figure 9D, lower panel), indicating that NF-κB element is essential for COX-2 promoter activity stimulated by S1P. These results further ensured that S1P-stimulated COX-2 promoter activity is mediated through enhancing interaction between NF-κB and the κB binging site of the COX-2 promoter region in HCFs.

We have found that S1P time-dependently induces PGE_2_ production (Figure 1D). Here, we further determined the involvement of these signaling components in S1P-induced PGE_2_ generation, as shown in Figure 9E, S1P-induced PGE_2_ generation was markedly attenuated by pretreatment with W123, CAY, GPA2A, PTX, Gö6976, PD98059, SB202190, SP600125, or helenalin. These results also demonstrated that S1PR1/3-mediated activation of PKCα/MAPKs and NF-κB by S1P is essential for COX-2 gene expression and PGE_2_ production in HCFs.

## Discussion

**FIGURE 10:**
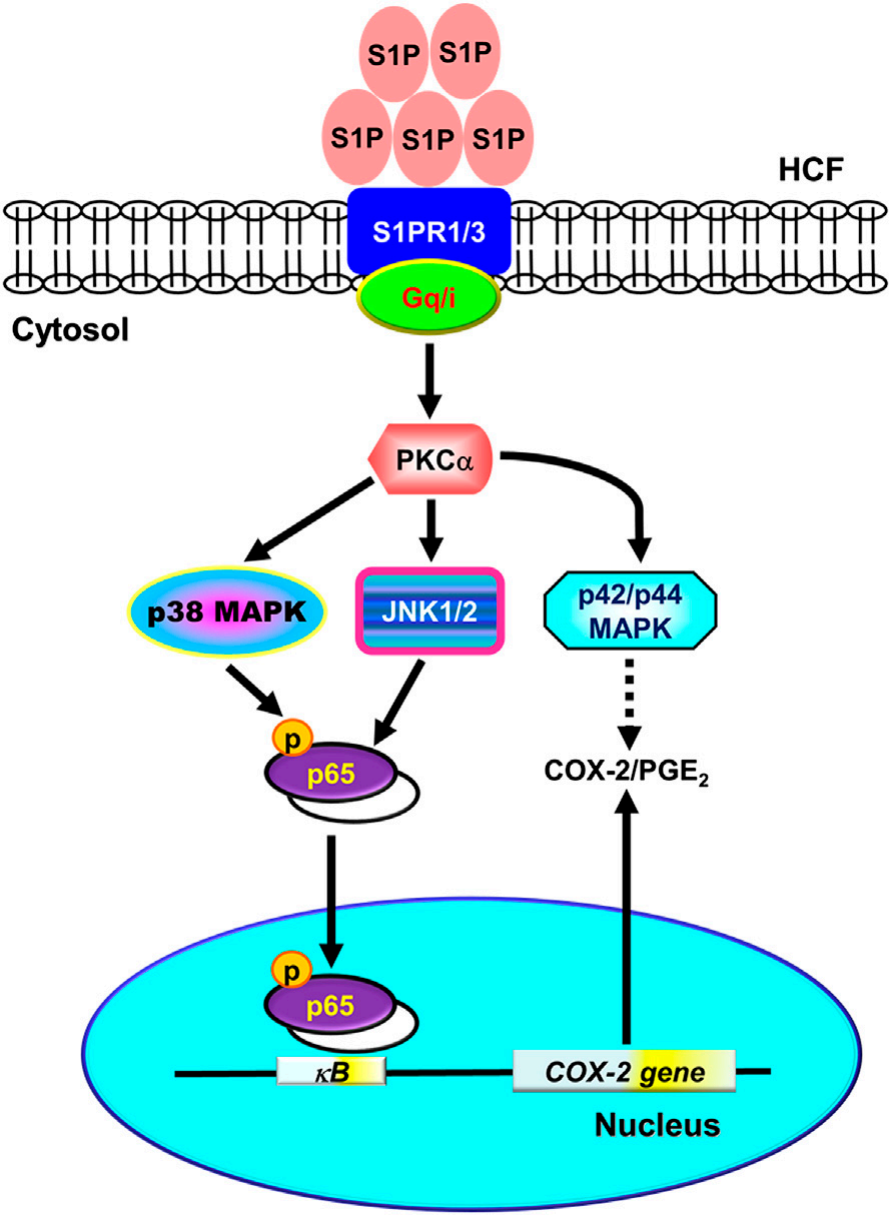
Schematic representation of signaling pathways involved in S1P-induced COX-2/PGE_2_ expression in HCFs. Binding of S1P to G_q_- or PTX-sensitive G_i_ protein-coupled S1PR1/3 results in PKCα-dependent phosphorylation of MAPKs (i.e., p42/p44 MAPK, p38 MAPK, and JNK1/2) leading to activation of NF-κB. The COX-2 transcription is dependently regulated by MAPK-mediated NF-κB cascades. These signaling pathways contribute to d activation of NF-κB required for COX-2 expression and PGE_2_ generation in HCFs.

Several inflammatory diseases of different tissues increase the expression of COX-2 that displays a wide range of biological functions, including development, proliferation, cancer, and inflammation (Williams et al., 1999). In these inflammatory responses, high levels of PGs (PGE_2_ in particular) synthesized by COX-2 are involved. Moreover, S1P is elevated in the regions of tissue injuries and inflammation (Ammit et al., 2001; Kitano et al., 2006; Takuwa et al., 2008; Watson et al., 2012). S1P has been shown to regulate the activity of COX-2 *via* MAPKs in various cell types (Davaille et al., 2000; Kim et al., 2003). Although some evidence implied that SIP possesses cellular protective function in the models of heart disease, these results predominantly showed the effects of SIP on either myocardiocyte or animal models. For example, Gomez et al. (2011) indicated that preconditioning of ischemia/reperfusion (I/R) activates second isoform of sphingosine kinase (SphK2) to produce mitochondrial S1P, leading to protective modulation via inhibition of mitochondrial permeability transition pore (PTP) opening in *sphk2* ^−/−^ mice (Gomez et al., 2011). Karliner et al. (2001) found S1P has cardioprotective effects against hypoxic injury in neonatal rat ventricular myocytes through signaling mechanisms involving PKC and mitochondrial K_ATP_ channels (Karliner et al., 2001). Theilmeier et al. (2006) using S1P-containing HDL to treat an *in vivo* mouse model of myocardial ischemia/reperfusion demonstrated that S1P protects the heart from ischemia/reperfusion injury *via* an S1P_3_-mediated and NO-dependent pathway (Theilmeier et al., 2006). Duan et al. (2007) revealed that the application of adenovirus-SPK1 gene injection could efficiently prevent I/R-induced myocardial injury, preserve cardiac function, and attenuate postischemic heart remodeling in a ligation model of rats (Duan et al., 2007). Most recently, Santos-Gallego et al. (2016) demonstrated that fingolimod, a S1P receptor agonist, could reduce infarct size in an acute MI animal model *via* attenuating phosphorylation of ERK1/2 and Akt signaling pathways (Santos-Gallego et al., 2016). These contrary effects may result from different types of cells, species, and experiment conditions. In the heart, the cardiac ﬁbroblasts express three types of S1PR, including S1PR_1-3_ (Landeen et al., 2007; Ahmed et al., 2017). Activation of cardiac S1PRs by S1P has been reported to affect cardiac contractility and heart rate, and induce heart diseases (Means and Brown, 2009; Porter and Turner, 2009). However, the molecular mechanisms underlying S1P-induced COX-2 expression were not completely defined in HCFs. In this study, we applied Western blot, RT-PCR, selective pharmacological inhibitors, transfection with siRNA, immunofluorescent staining, and promoter analyses to investigate the mechanisms of COX-2 expression induced by S1P. Our results demonstrated that in HCFs, activation of S1PR_1/3_-mediated PKCα-dependent MAPKs (p42/p44 MAPK, p38 MAPK, and JNK1/2), and NF-κB signaling pathway is essential for S1P-induced COX-2 gene expression and PGE_2_ production (Figure 10).

To date, S1P is known to activate S1P receptors (S1PR_1-5_), a heterotrimeric GPCR, which stimulates multiple signaling pathways and regulates diverse cellular functions (Means and Brown, 2009; Aarthi et al., 2011). The predominant pathways activated by S1P are mediated through S1PR coupling to G_q_ or G_i_ protein, resulting in activating PLC-β and further hydrolysis of PI, and finally leading to Ca^2+^ accumulation and activating PKC (Takuwa et al., 2008). Here we demonstrated that S1P recruits S1PR_1/3_ coupling to either G_q_ or PTX-sensitive G_i_ protein to induce COX-2 expression in HCFs. Subsequently, we further investigated the role of PKCs in S1P-induced COX-2 expression. PKC represents a family of 12 phospholipid-dependent Ser/Thr kinases that participate in diverse pathways to modulate several cellular functions including cell proliferation, apoptosis, and stress responsiveness. Nodai et al. have indicated that S1P induces COX-2 expression in rat VSMCs through Ca^2+^-dependent PKC activation via S1PR_3_ coupling to G protein either PTX-sensitive or PTX -insensitive (Nodai et al., 2007). In addition, S1P enhances the IL-1β-induced COX-2 expression *via* PKC activation that may contribute to intestinal inflammation in mouse intestinal subepithelial myofibroblasts (Ohama et al., 2008). These reports indicate that PKC signaling is involved in COX-2 induction in various types of cells. In HCFs, the data showed that pretreatment with PKCα inhibitor Gö6976 markedly attenuated S1P-induced COX-2 expression and PGE_2_ generation. Moreover, we also determined that S1P stimulates PKCα translocation *via* S1PR_1/3_ coupling to G_q_ or PTX-sensitive G_i_ protein cascades. These results suggested that activation of PKCα by S1P is critical for COX-2 expression in HCFs.

Increasing evidence indicates that GPCR agonists activate MAPKs through diverse signaling pathways (Marshall, 1994). MAPK cascades have been revealed to act as an important moderator of S1P in different cell types (Pyne and Pyne, 2000; Tanimoto et al., 2004). In a variety of injury and inflammation, the expression of inflammatory genes might be mediated through MAPKs activation (Davaille et al., 2000; Kim et al., 2003; Hsieh et al., 2007). Our previous report has demonstrated that activation of p42/p44 MAPK involves in the S1P-induced COX-2 expression in VSMCs (Hsieh et al., 2006). In this study, we further confirmed the roles of MAPKs involved in the S1P-induced COX-2 expression in HCFs. The selective inhibitors of MEK1/2 (PD98059), p38 MAPK (SB202190), and JNK1/2 (SP600125) significantly attenuated S1P-induced COX-2 mRNA and protein expression and their phosphorylation, suggesting that activation of these MAPKs is necessary for S1P-induced COX-2 expression in HCFs. In addition, transfection with siRNA of respective MAPKs (i.e., p42, p38, and JNK1) markedly reduced COX-2 expression induced by S1P, confirming the roles of MAPKs in COX-2 expression. These results are align with the reports showing that activation of MAPKs plays a pivotal role in the up-regulation of COX-2 in various cell types (Kim et al., 2003; Volzke et al., 2014). However, in rat VSMCs, MAPK activation seems to be not required for S1P-induced COX-2 induction (Nodai et al., 2007). Cell type-specific or different experimental conditions may cause these differences.

It was worth noting that activation of S1P receptors relays the signaling through sequential components which may be also modulated by the other components. Thus, the final effects on phosphorylation of protein kinases such as p42/p44 MAPK, p38 MAPK, and JNK1/2 were not completely attenuated by the pharmacological inhibitors, since only one inhibitor was applied to observe its effect on phosphorylation of each protein kinase stimulated by S1P. Thus, the activation of p42/p44 MAPK, p38 MAPK, and JNK1/2 was still occurring in the presence of different inhibitors. The other reason for the still preserved activity of each component may be multiple upstream signaling pathways to regulate the downstream signalings. These results could provide the evidence for the phosphorylation of protein kinases mediated through S1P receptor subtypes, G protein, and PKCs in HCFs challenged with S1P. Indeed, the combining effects of different S1P receptor subtypes such as S1PR1 and three on the expression of COX-2 were investigated in our study (Supplementary Figure S1). Pretreatment with W123 (1 μM) plus CAY10444 (0.3 μM) synergistically attenuated the S1P-induced COX-2 expression being more than that of pretreatment with either CAY10444 or W123 alone. The combinatory effects on phosphorylation of protein kinases leading to the expression of inflammatory proteins could be an important issue for further study in these cells.

It has been well recognized that inflammatory responses triggered by extracellular stimuli are highly dependent on activation of the transcription factor NF-κB, which plays a key role in the expression of several genes (Barnes and Karin, 1997). The 5′-flanking region of the COX-2 promoter contains several binding sequences of various transcription factors such as NF-κB (Tanabe and Tohnai, 2002). Therefore, aberrant activation of several distinct transcription factors dependent on agonists may regulate the COX-2 transcription (Kim and Fischer, 1998; Hsieh et al., 2007). These studies suggest that NF-κB is involved in the regulation of COX-2 expression in the development of the inflammatory responses. Our data showed that S1P-induced COX-2 gene expression and PGE_2_ generation was significantly abolished by a selective NF-κB inhibitor helenalin or NF-κB p65 siRNA, suggesting that NF-κB (p65) is involved in S1P-induced COX-2 expression in HCFs. Moreover, S1P-stimulated p65 NF-κB translocation, binding to COX-2 promoter region, and NF-κB transcriptional activity was significantly inhibited by helenalin and the MAPK inhibitors including SB202190 (p38 MAPK) or SP600125 (JNK1/2), but not PD98059 (MEK1/2), suggesting that NF-κB activated by S1P is mediated through p38 MAPK- and JNK1/2-dependent mechanisms in HCFs. Interestingly, in HCFs, the p42/p44 MAPK is not involved in activation of NF-κB by S1P, consistent with our previous report indicating that activation of NF-κB by S1P independent of p42/p44 MAPK in rat VSMCs (Hsieh et al., 2006). It is worth noting that how p42/p44 MAPK mediated the S1P-induced COX-2 expression is preserved for future study. Our data further showed that S1P-stimulated NF-κB translocation and transcriptional activity was significantly attenuated by blocking G_q_ and PTX-sensitive G_i_ protein-coupled S1PR1/3-mediated activation of PKCα cascades, indicating that S1P-induced activation of NF-κB is mediated through S1PR1/3-dependent activation of PKCα linking to p38 MAPK and JNK1/2 pathways. These findings are consistent with previous studies indicating that COX-2 expression and prostacyclin release induced by thrombin or ET-1 are mediated through MAPKs and NF-κB activation in endothelial cells (Syeda et al., 2006; Lin et al., 2013). For the roles of these signaling molecules, we confirmed that MAPKs-dependent activation of NF-κB plays a critical role in the up-regulation of COX-2/PGE_2_ induced by S1P in HCFs.

In conclusion, we reported here that the S1P/S1PR system exerted its stimulatory effects on COX-2 gene expression in HCFs. The G_q_ and PTX-sensitive G_i_ protein-coupled S1PR1/3, PKCα, MAPKs, and NF-κB signaling pathways cooperatively implicate in these effects of S1P. Based on pieces of literature and our results, Figure 10 depicts the signaling mechanisms underlying S1P-induced COX-2 gene expression in HCFs. These findings in relation to S1P-induced COX-2 expression and PGE_2_ production imply that S1P/S1PR system might play an important role in heart inflammatory disorders mediated through PKCα, MAPKs (i.e., p42/p44 MAPK, p38 MAPK, JNK1/2), and NF-κB signaling pathways in HCFs. These results implicate a role for cardiac fibroblasts, in addition to their organized and preservative functions, as inflammatory cells involved in the production of chemical mediators, which may contribute to the inflammatory responses in heart diseases. Although the limitation of the present study is the lack of *in vivo* evidence to support our findings, it is worth further conducting experiments using animal models. Overall these findings imply that S1P might play a crucial role in the pathogenesis of heart inflammatory diseases and provide useful insights for developing effective therapeutic targets in heart diseases.

## Data Availability Statement

The raw data supporting the conclusions of this manuscript will be made available by the authors, without undue reservation, to any qualified researcher.

## Author Contributions

C-CY, L-DH, M-HS, and C-MY substantially contributed to the conception or design of the work, the acquisition, analysis, and interpretation of data for the work. C-CY, L-DH, M-HS, and C-MY drafted the work and revised it critically for important intellectual content. C-CY, L-DH, M-HS, and C-MY finally approved the version to be published. C-CY, L-DH, M-HS, and C-MY agreed to be accountable for all aspects of the work in ensuring that questions related to the accuracy or integrity of any part of the work are appropriately investigated and resolved.

## Funding

This work was supported by the Ministry of Science and Technology, Taiwan [Grant numbers: MOST 107‐2320‐B‐182‐039‐071‐MY2, MOST108-2320-B-039-061, and MOST108-2320-B-182-014]; China Medical University, Taiwan [Grant numbers: CMU109-MF-08]; Chang Gung Medical Research Foundation, Taiwan [Grant numbers: CMRPG5F0203, CMRPG5I0041, CMRPG5J0141, CMRPG5J0142].

## Conflict of Interest

The authors declare that the research was conducted in the absence of any commercial or financial relationships that could be construed as a potential conflict of interest.

## Glossary

BCA: bicinchoninic acid;
ChIP: chromatin immunoprecipitation
COX-2: cyclooxygenase-2
ECL: enhanced chemiluminescence
FBS: fetal bovine serum
GPCRs: G protein-coupled receptors
HCFs: human cardiac fibroblasts
IP3: inositol trisphosphate
JNK1/2: c-Jun N-terminal kinase
MAPKs: mitogen-activated protein kinases
PTX: pertussis toxin
PI: phosphoinositide
PLCβ: phospholipase C β
PGE2: prostaglandin E2
PKC: protein kinase C
S1P: sphingosine 1-phosphate
