# Supplementary figures and images for: Sphingosine 1-Phosphate Induces Cyclooxygenase-2/Prostaglandin E2 Expression via PKCα-dependent Mitogen-Activated Protein Kinases and NF-κB Cascade in Human Cardiac Fibroblasts
Source: Front Pharmacol. 2020 Oct 30;11:569802. doi: 10.3389/fphar.2020.569802 (PMC7662885; doi:10.3389/fphar.2020.569802)

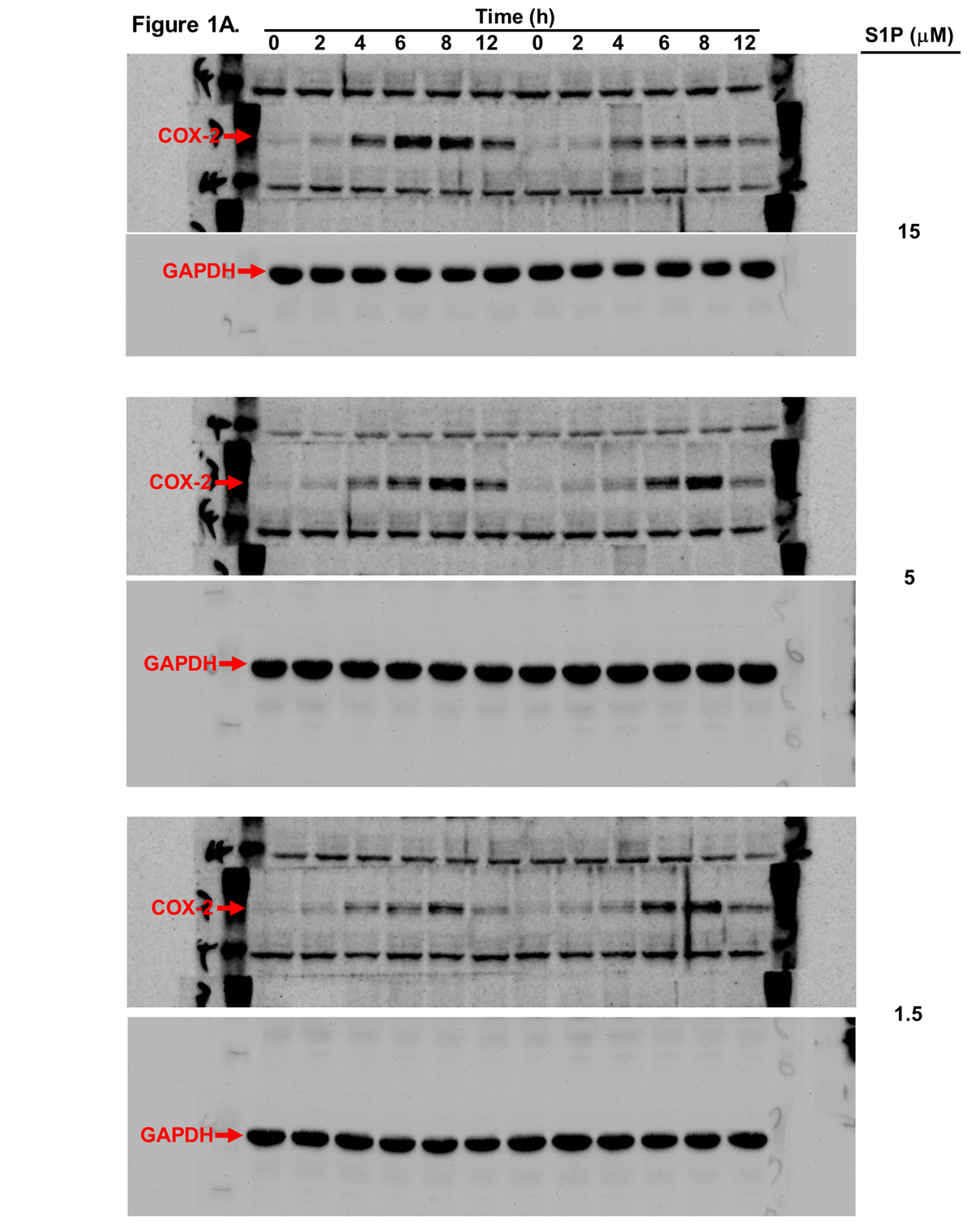

Supplement: Supplementary file 1 [file datasheet1.zip › Supplementary material/Figure 1A..tif]

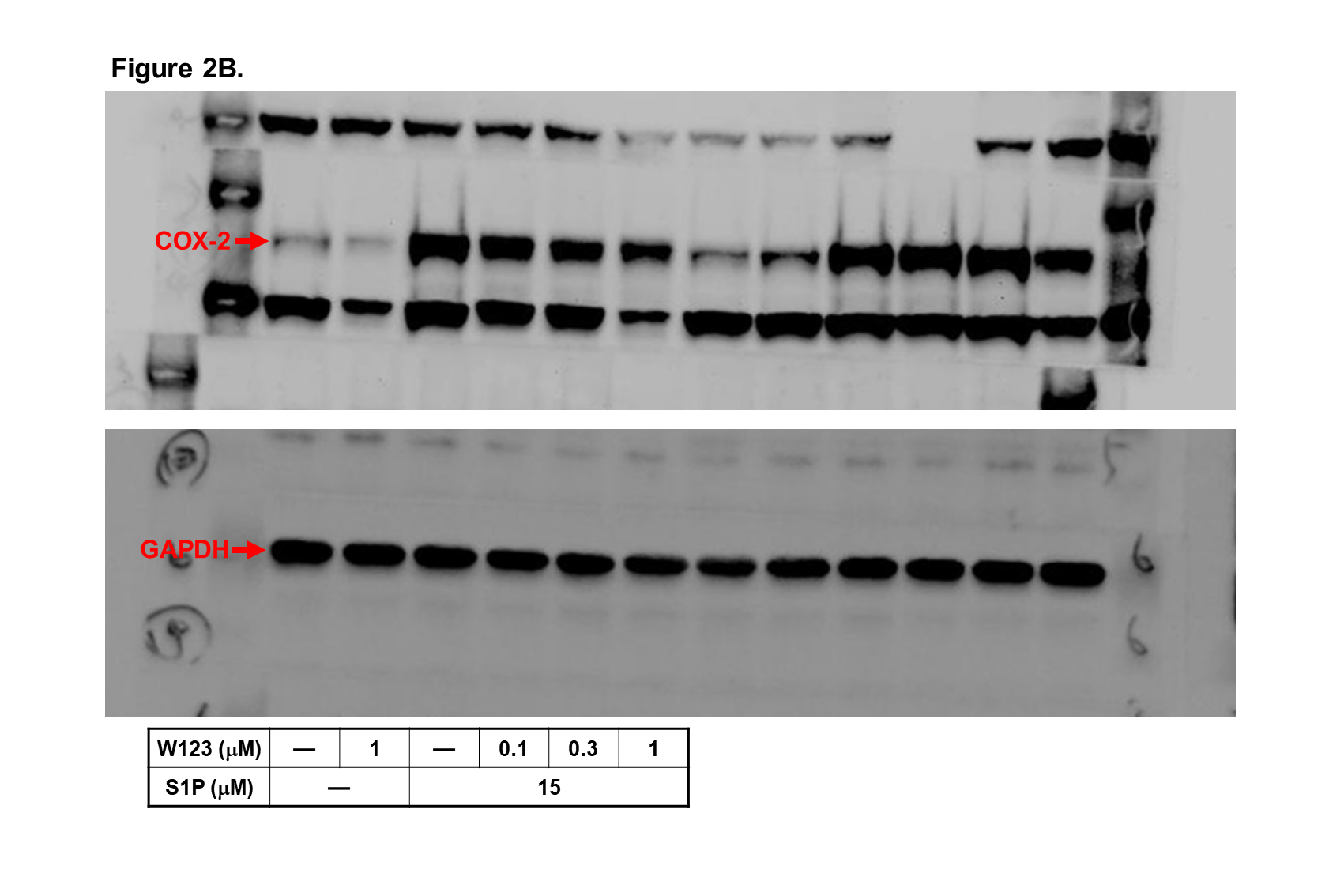

Supplement: Supplementary file 1 [file datasheet1.zip › Supplementary material/Figure 2B..tif]

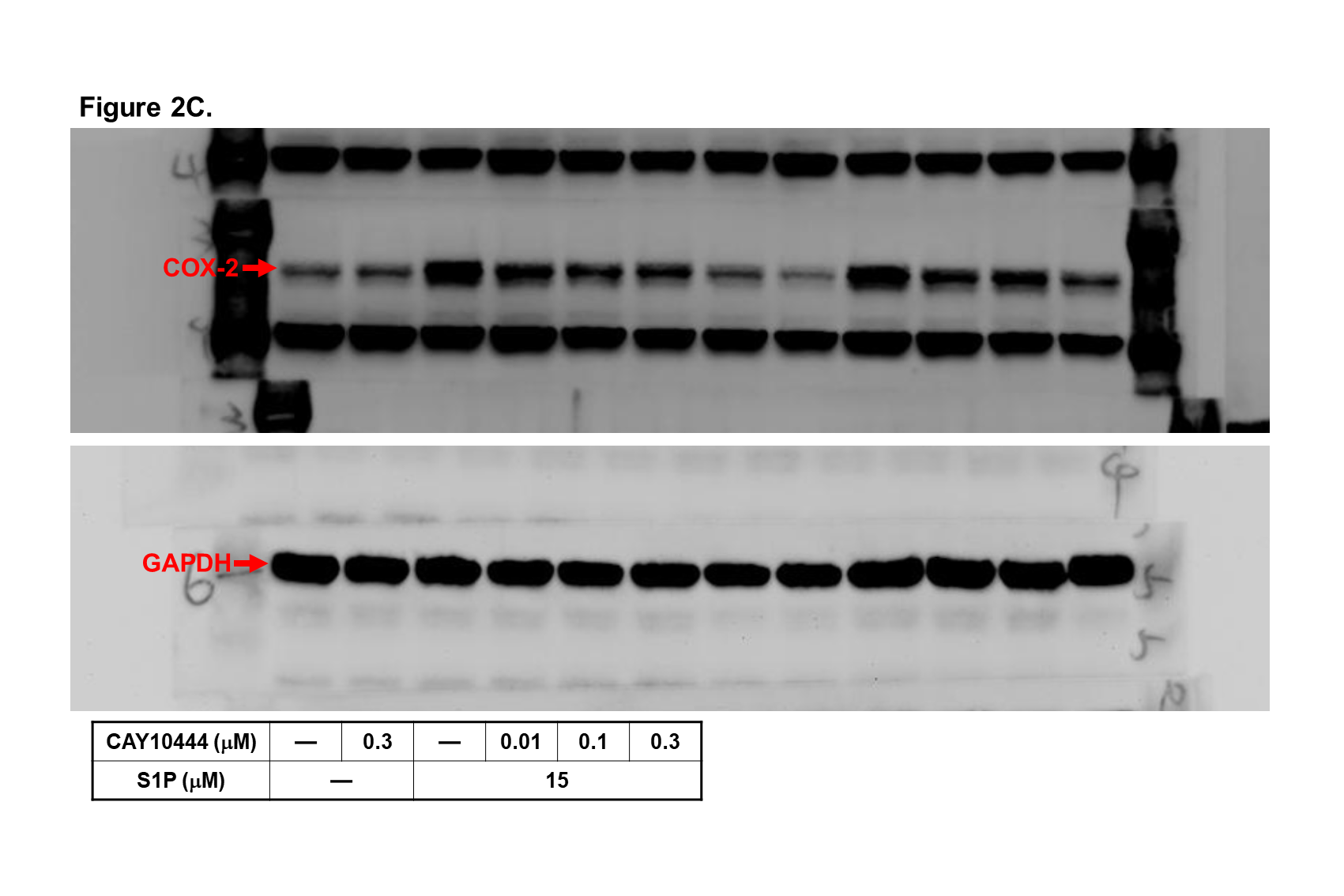

Supplement: Supplementary file 1 [file datasheet1.zip › Supplementary material/Figure 2C..tif]

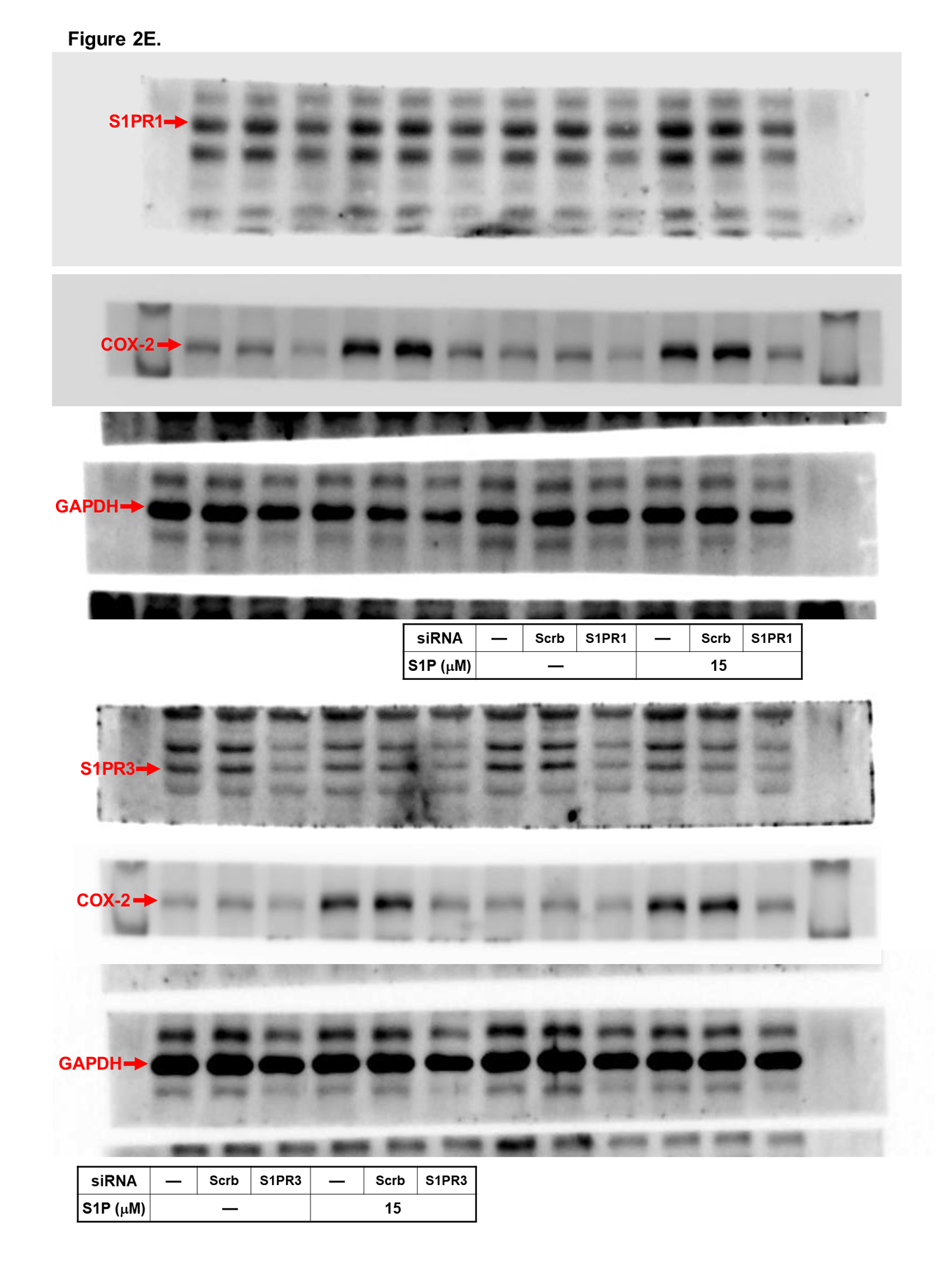

Supplement: Supplementary file 1 [file datasheet1.zip › Supplementary material/Figure 2E..tif]

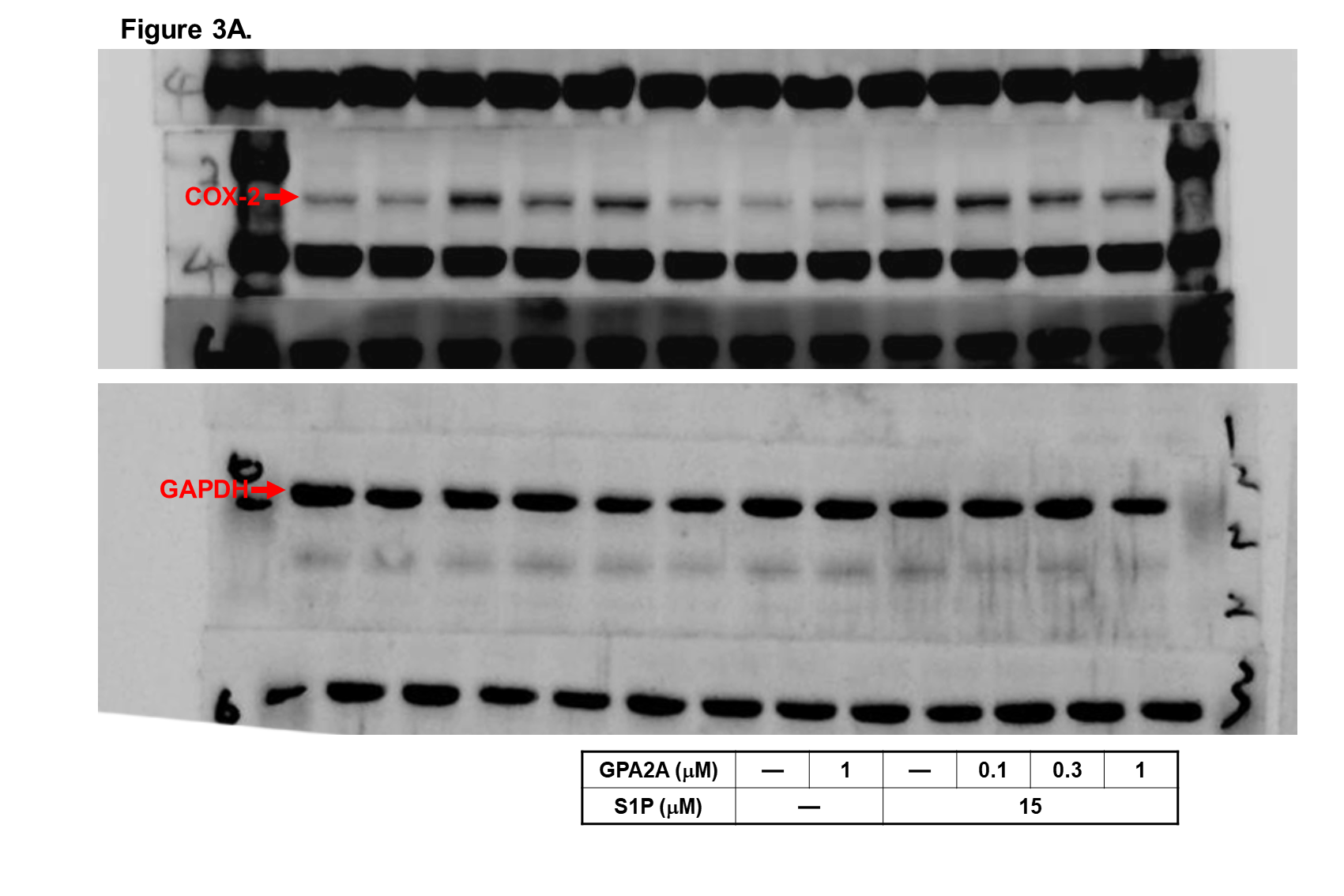

Supplement: Supplementary file 1 [file datasheet1.zip › Supplementary material/Figure 3A..tif]

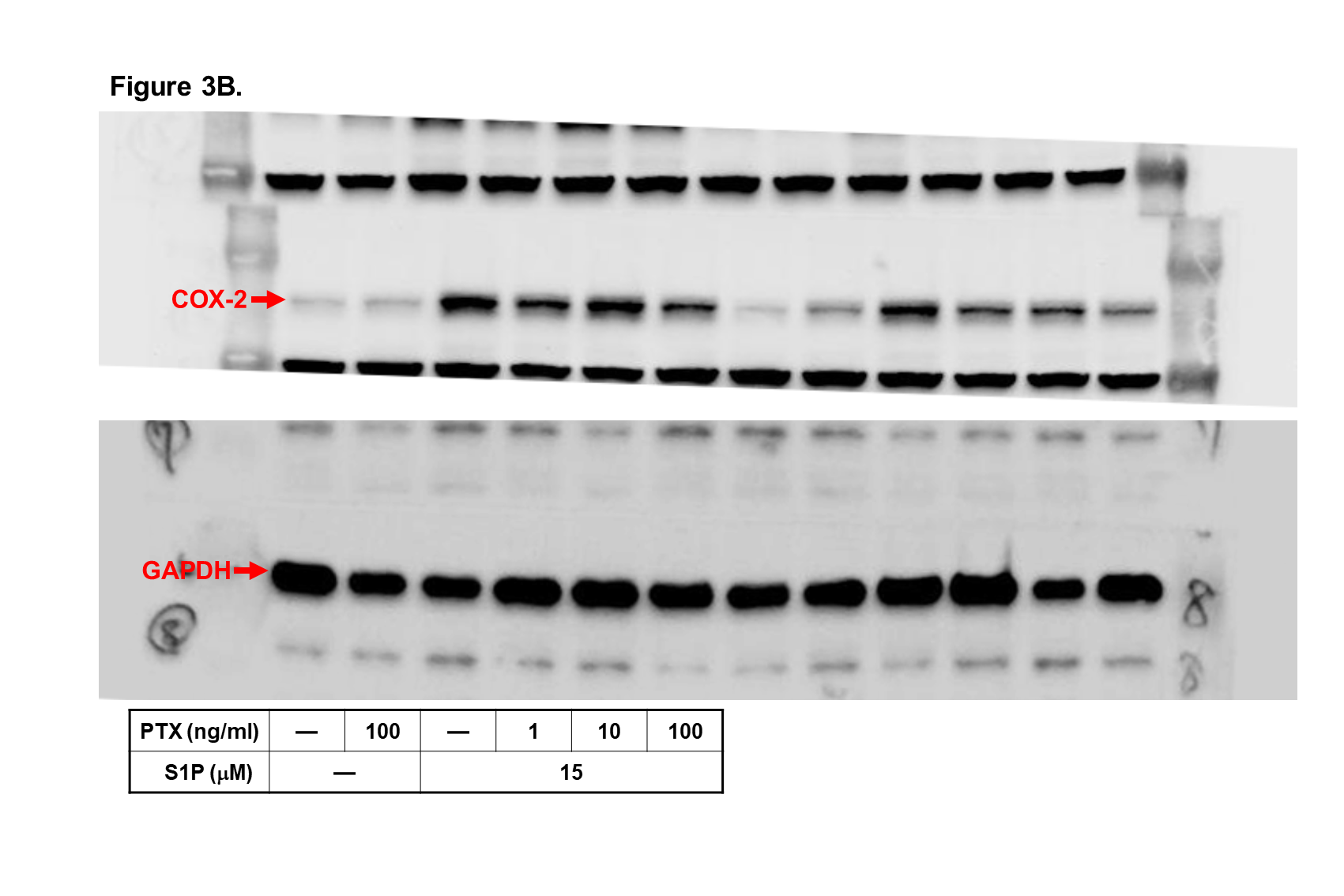

Supplement: Supplementary file 1 [file datasheet1.zip › Supplementary material/Figure 3B..tif]

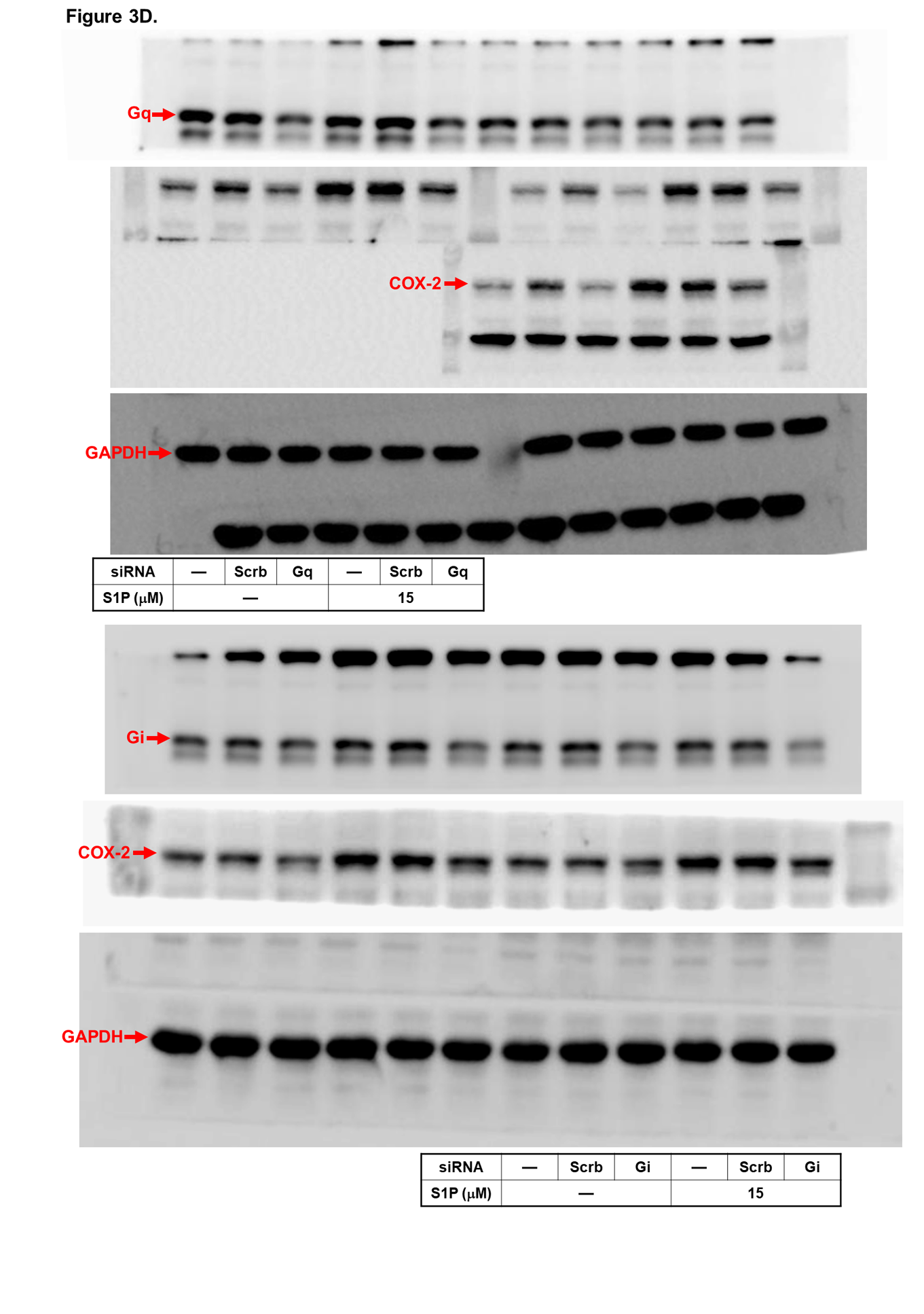

Supplement: Supplementary file 1 [file datasheet1.zip › Supplementary material/Figure 3D..tif]

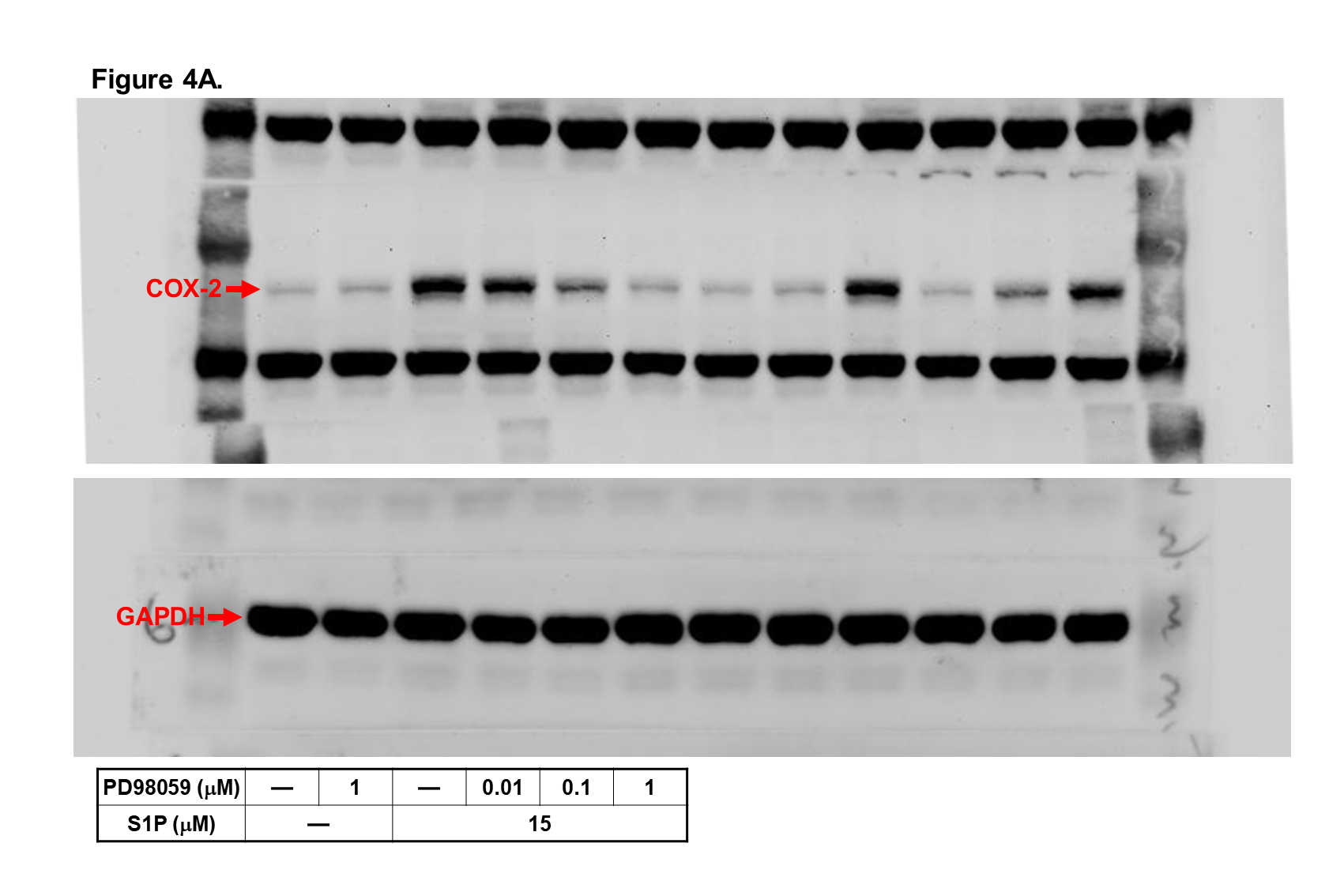

Supplement: Supplementary file 1 [file datasheet1.zip › Supplementary material/Figure 4A..tif]

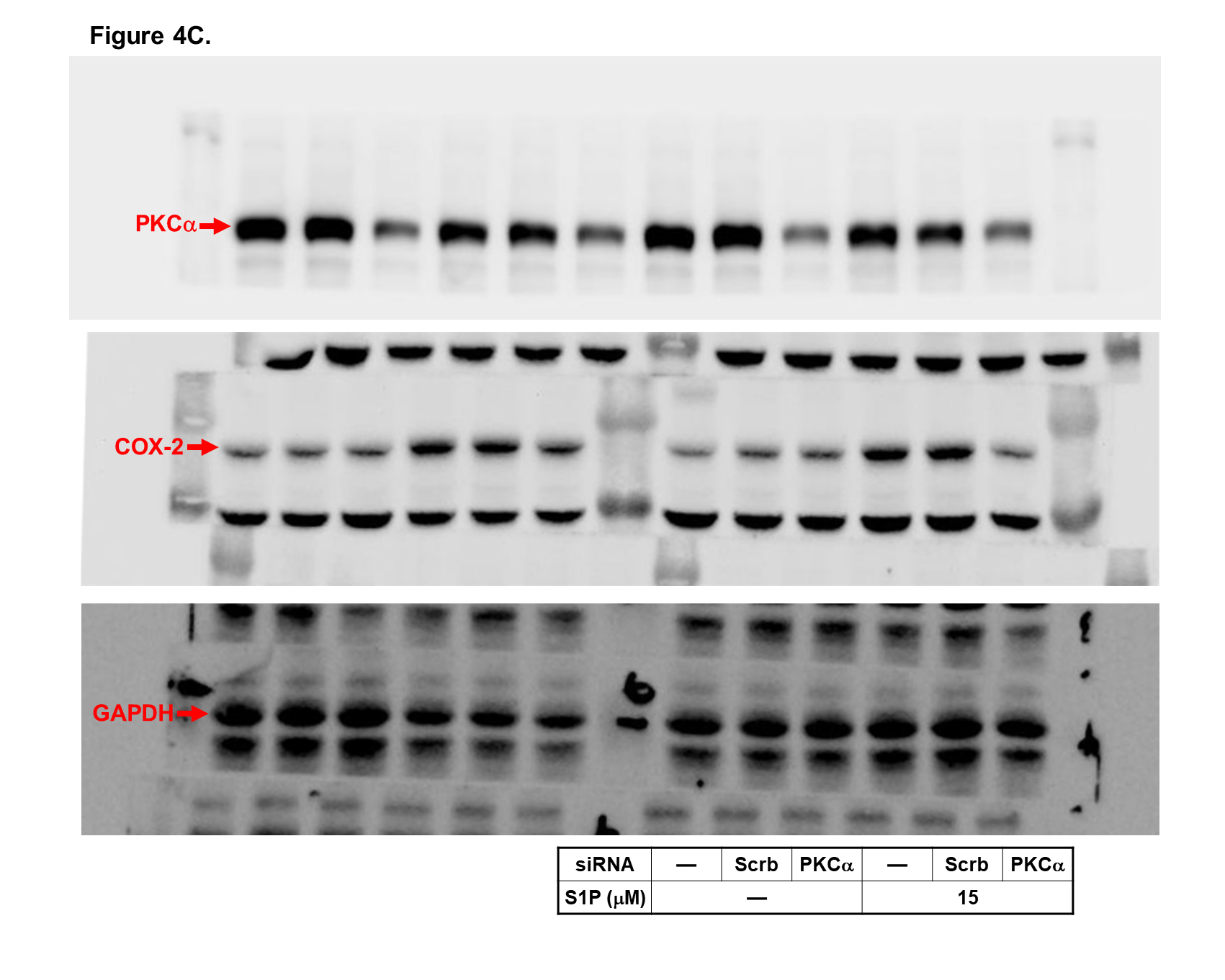

Supplement: Supplementary file 1 [file datasheet1.zip › Supplementary material/Figure 4C..tif]

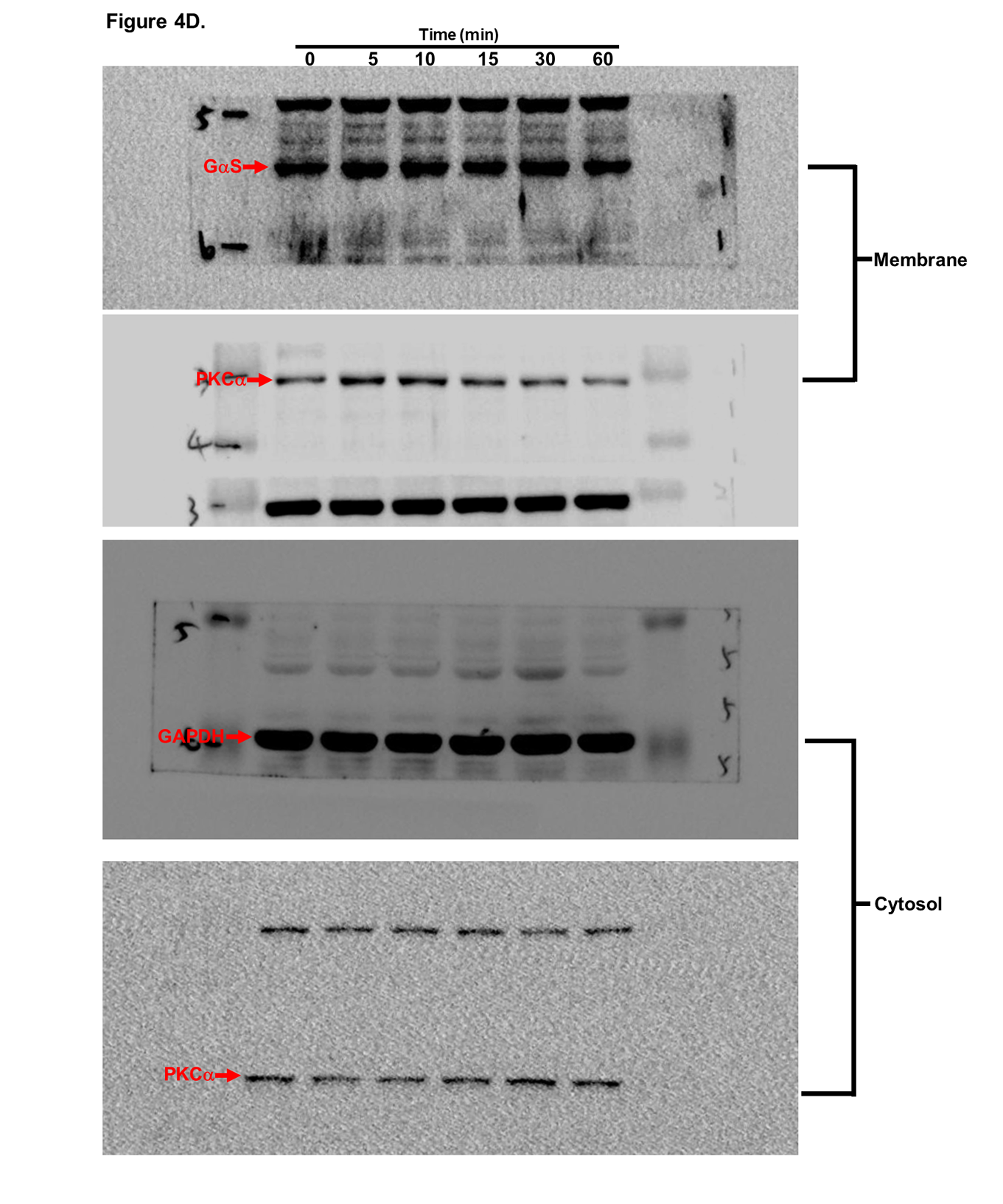

Supplement: Supplementary file 1 [file datasheet1.zip › Supplementary material/Figure 4D..tif]

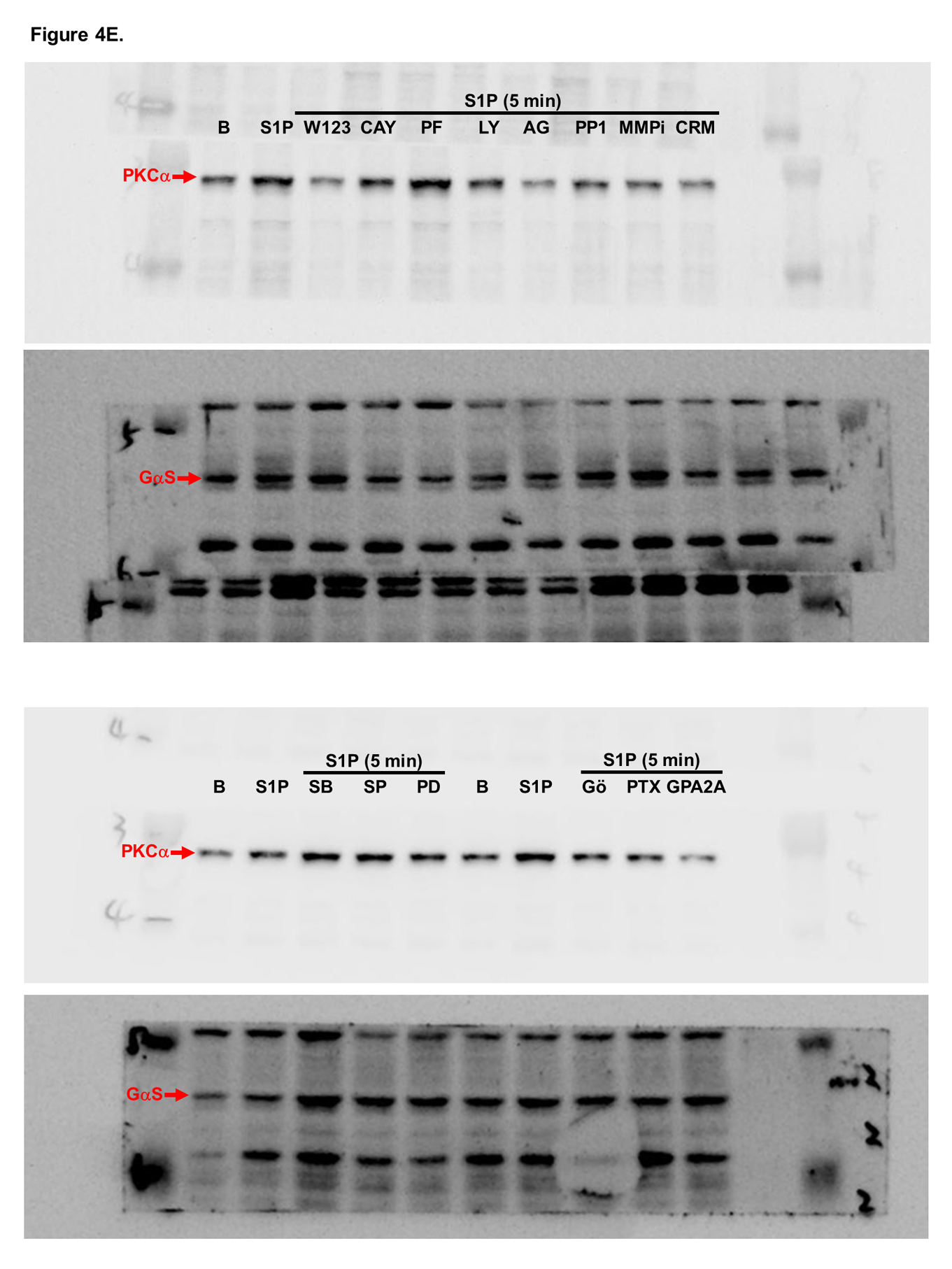

Supplement: Supplementary file 1 [file datasheet1.zip › Supplementary material/Figure 4E..tif]

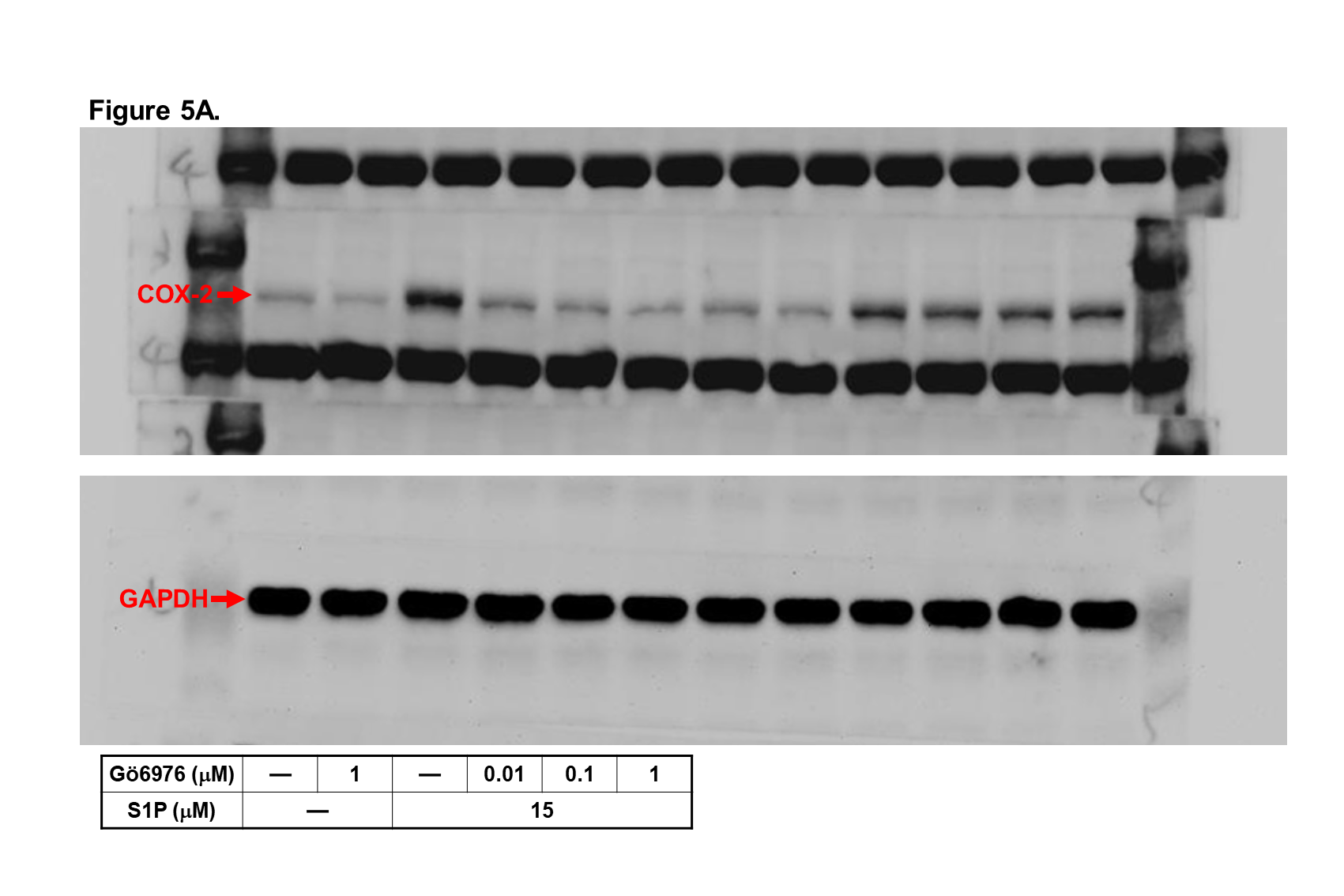

Supplement: Supplementary file 1 [file datasheet1.zip › Supplementary material/Figure 5A..tif]

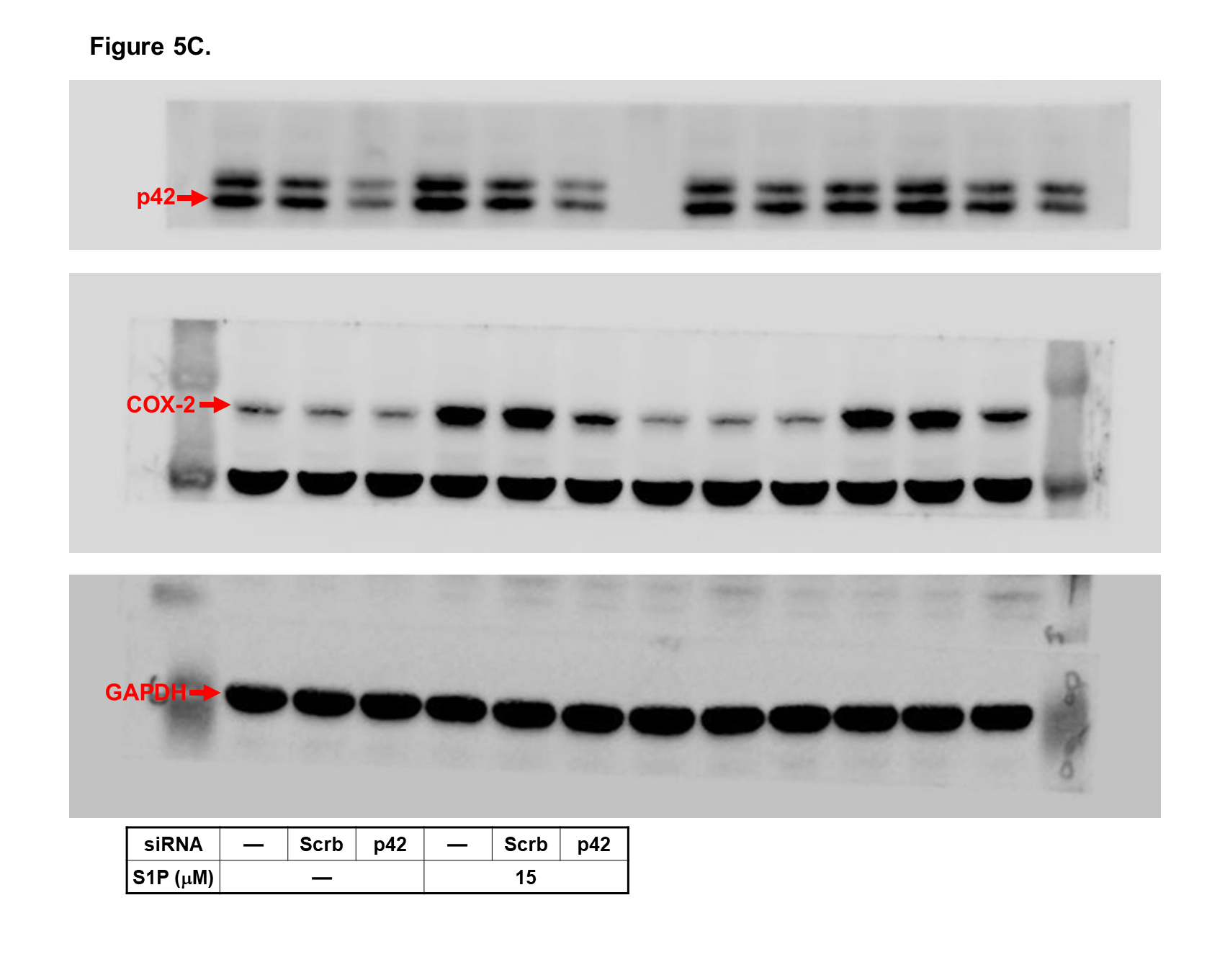

Supplement: Supplementary file 1 [file datasheet1.zip › Supplementary material/Figure 5C..tif]

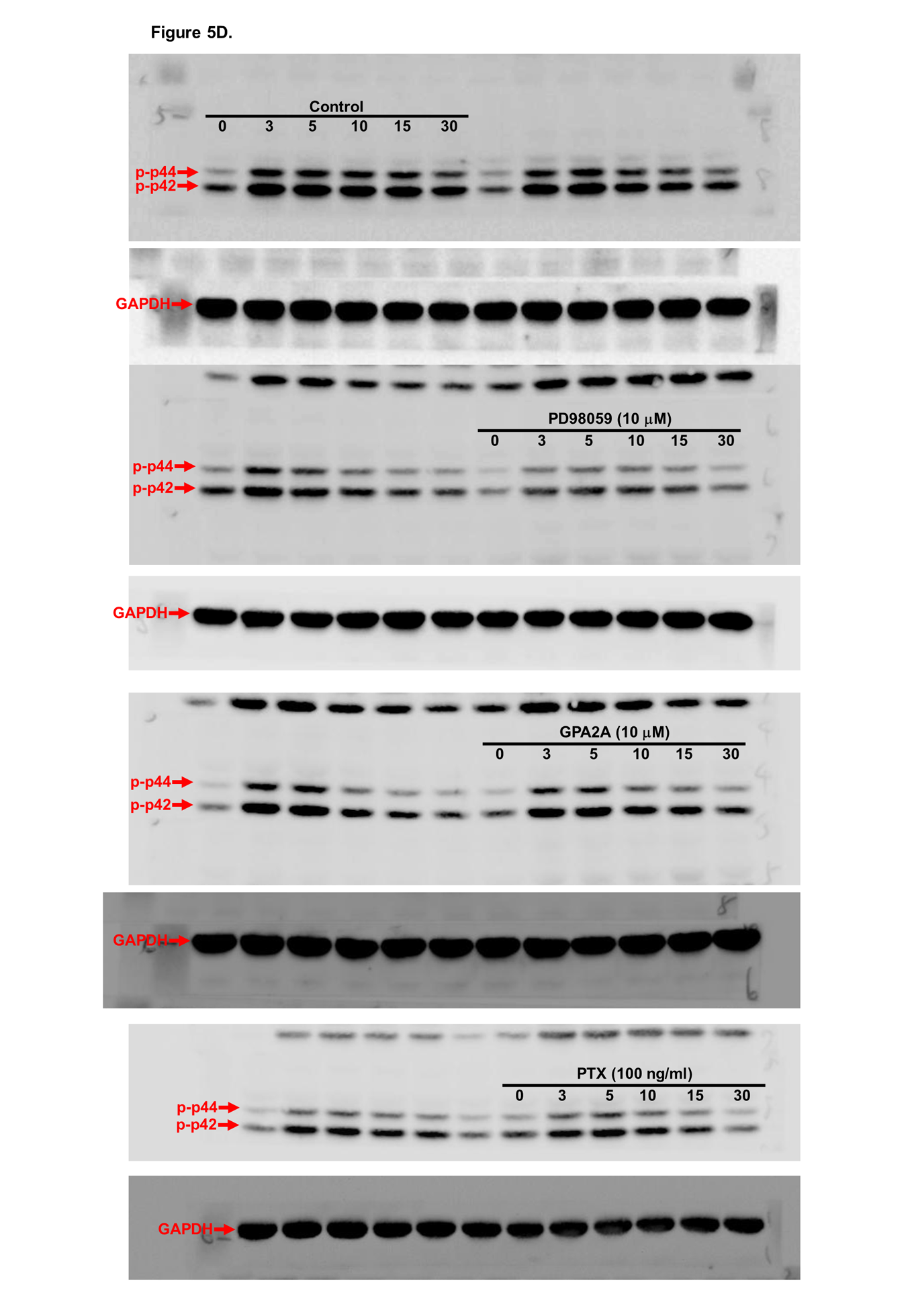

Supplement: Supplementary file 1 [file datasheet1.zip › Supplementary material/Figure 5D-1..tif]

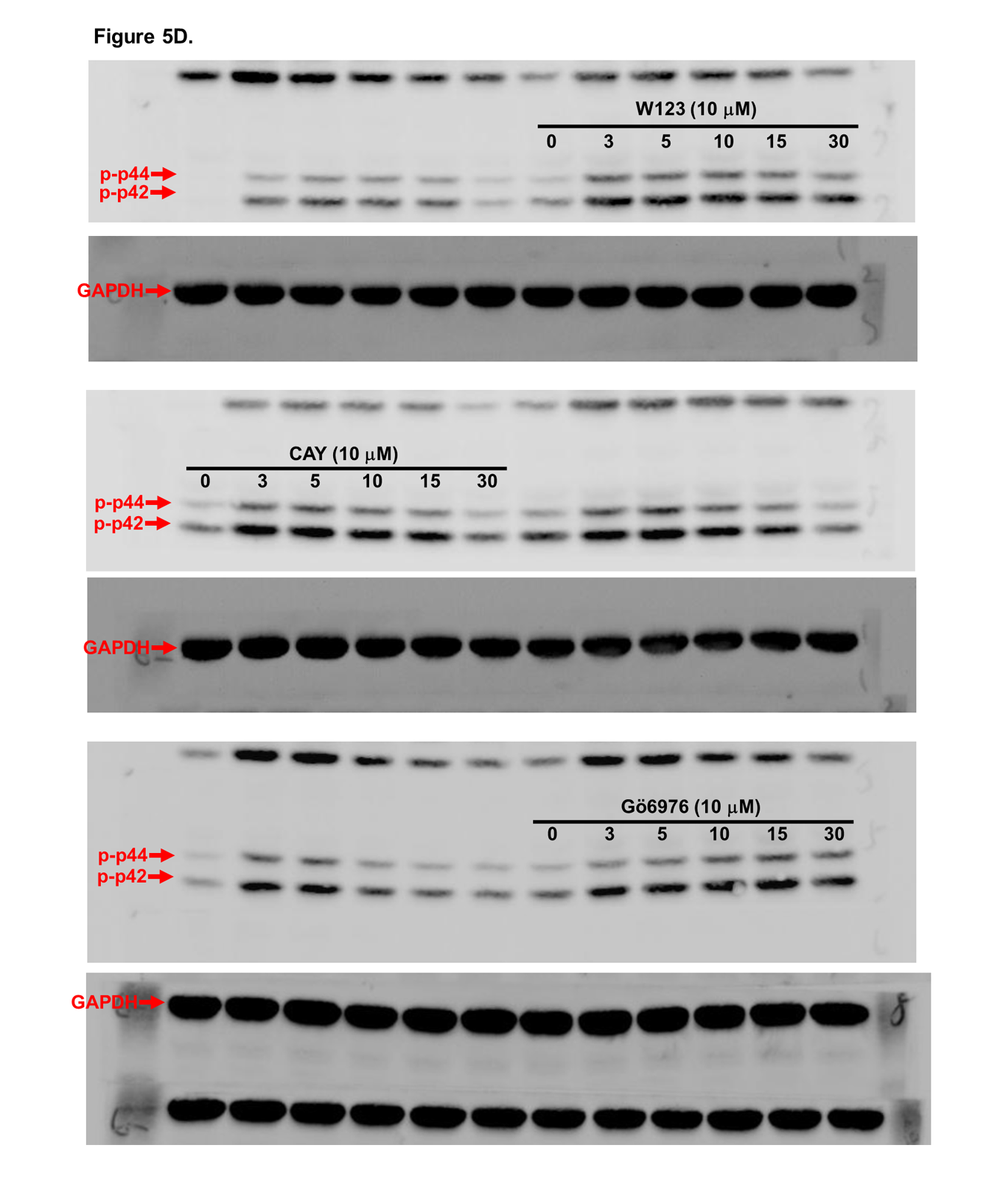

Supplement: Supplementary file 1 [file datasheet1.zip › Supplementary material/Figure 5D-2..tif]

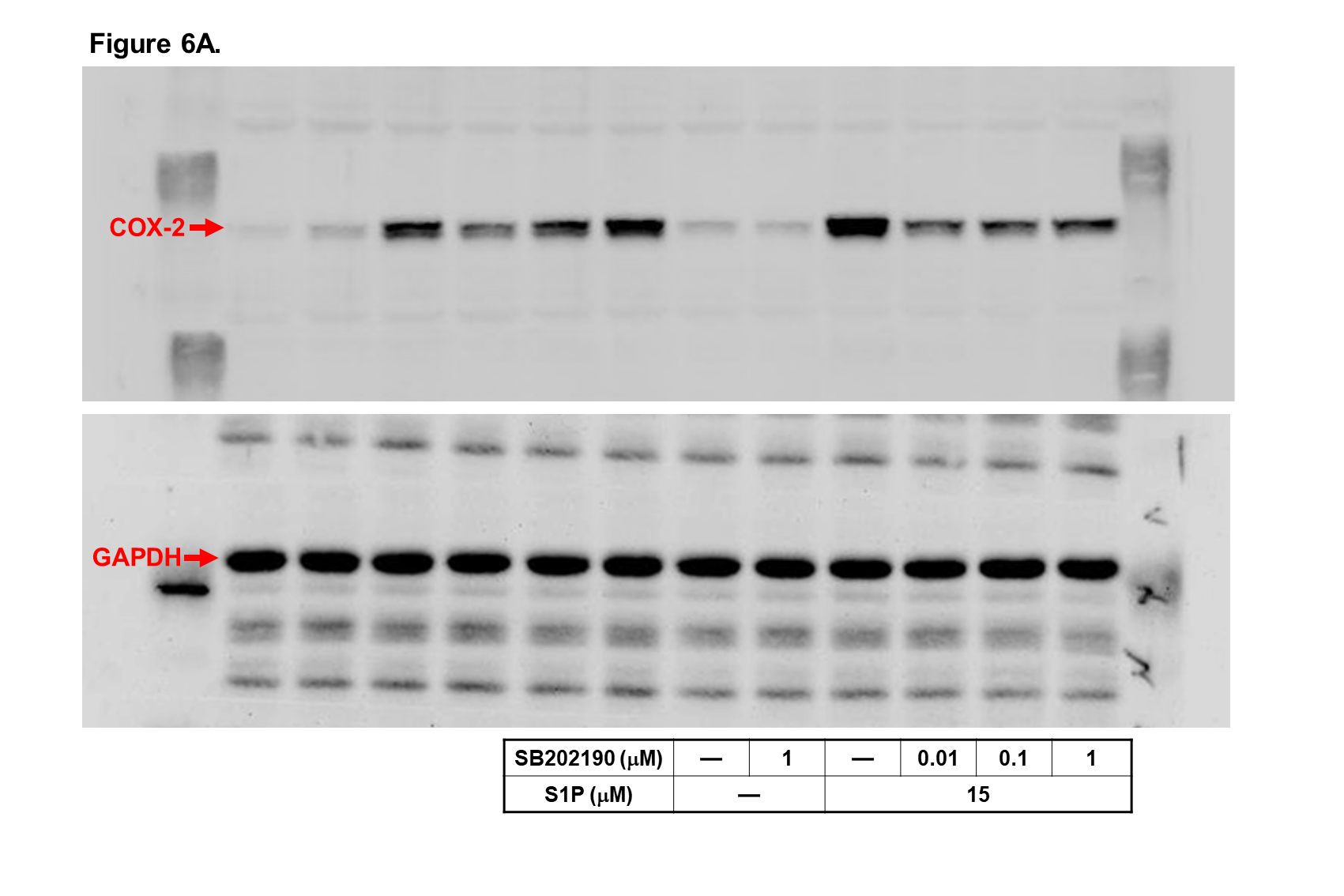

Supplement: Supplementary file 1 [file datasheet1.zip › Supplementary material/Figure 6A..tif]

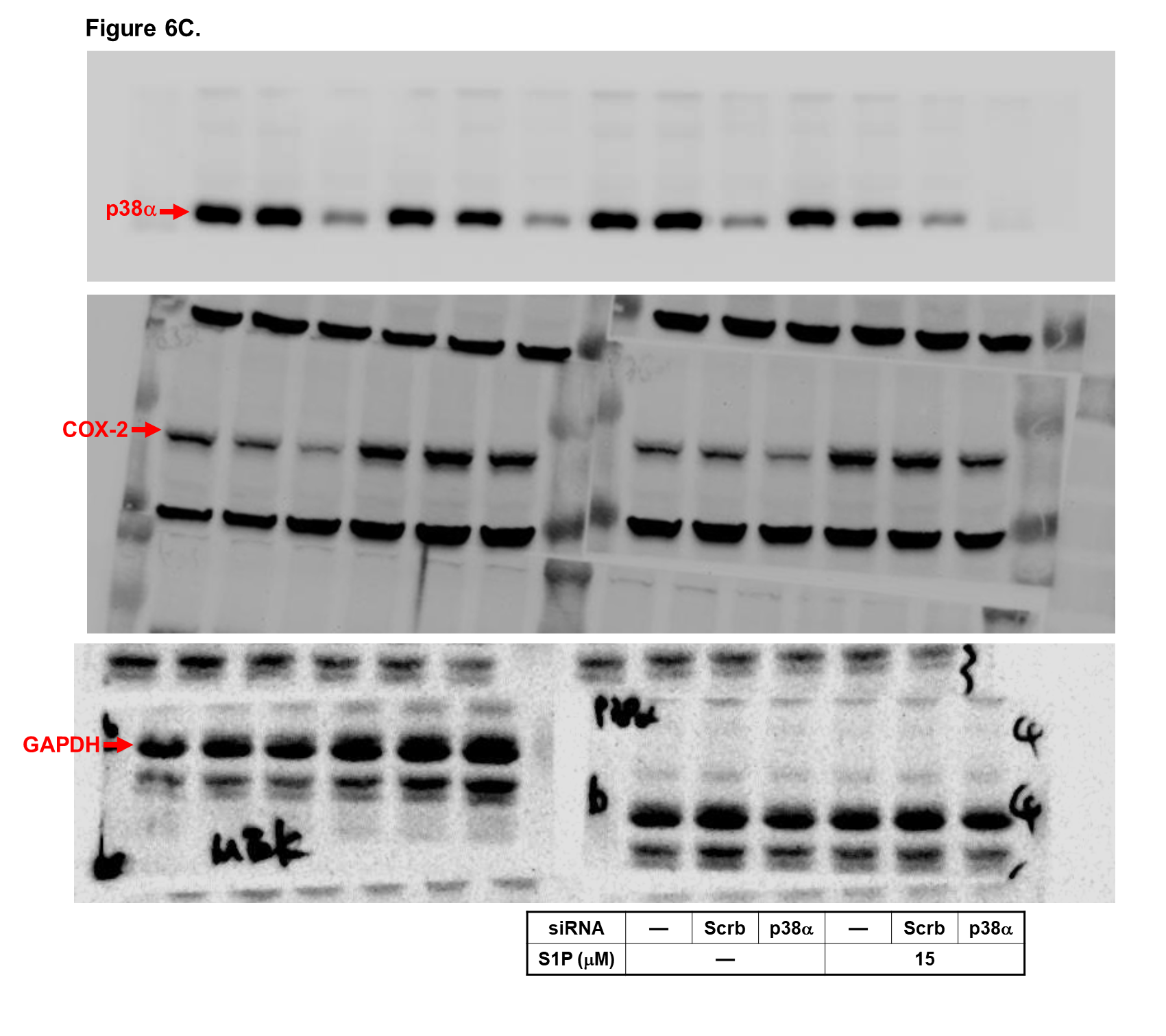

Supplement: Supplementary file 1 [file datasheet1.zip › Supplementary material/Figure 6C..tif]

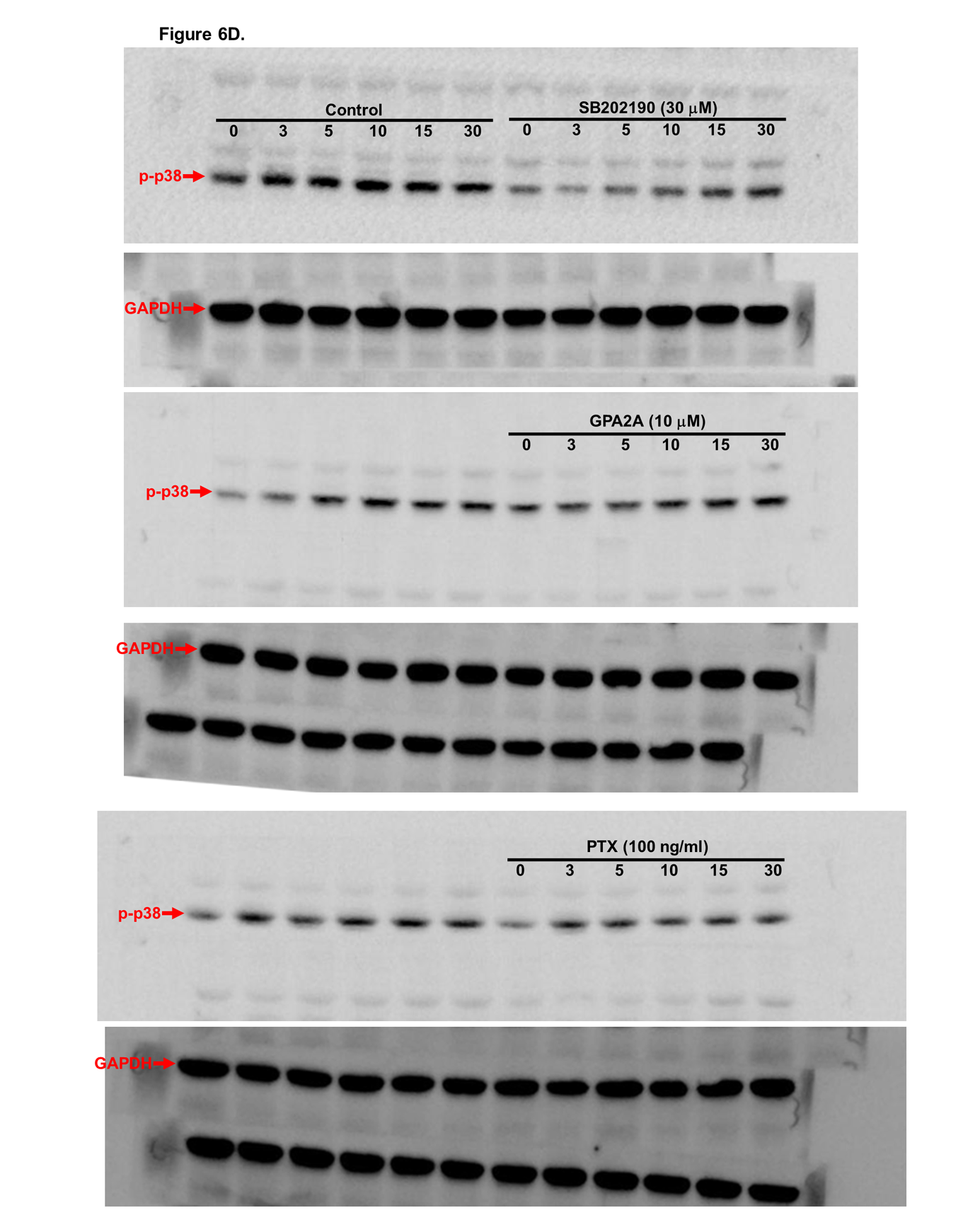

Supplement: Supplementary file 1 [file datasheet1.zip › Supplementary material/Figure 6D-1..tif]

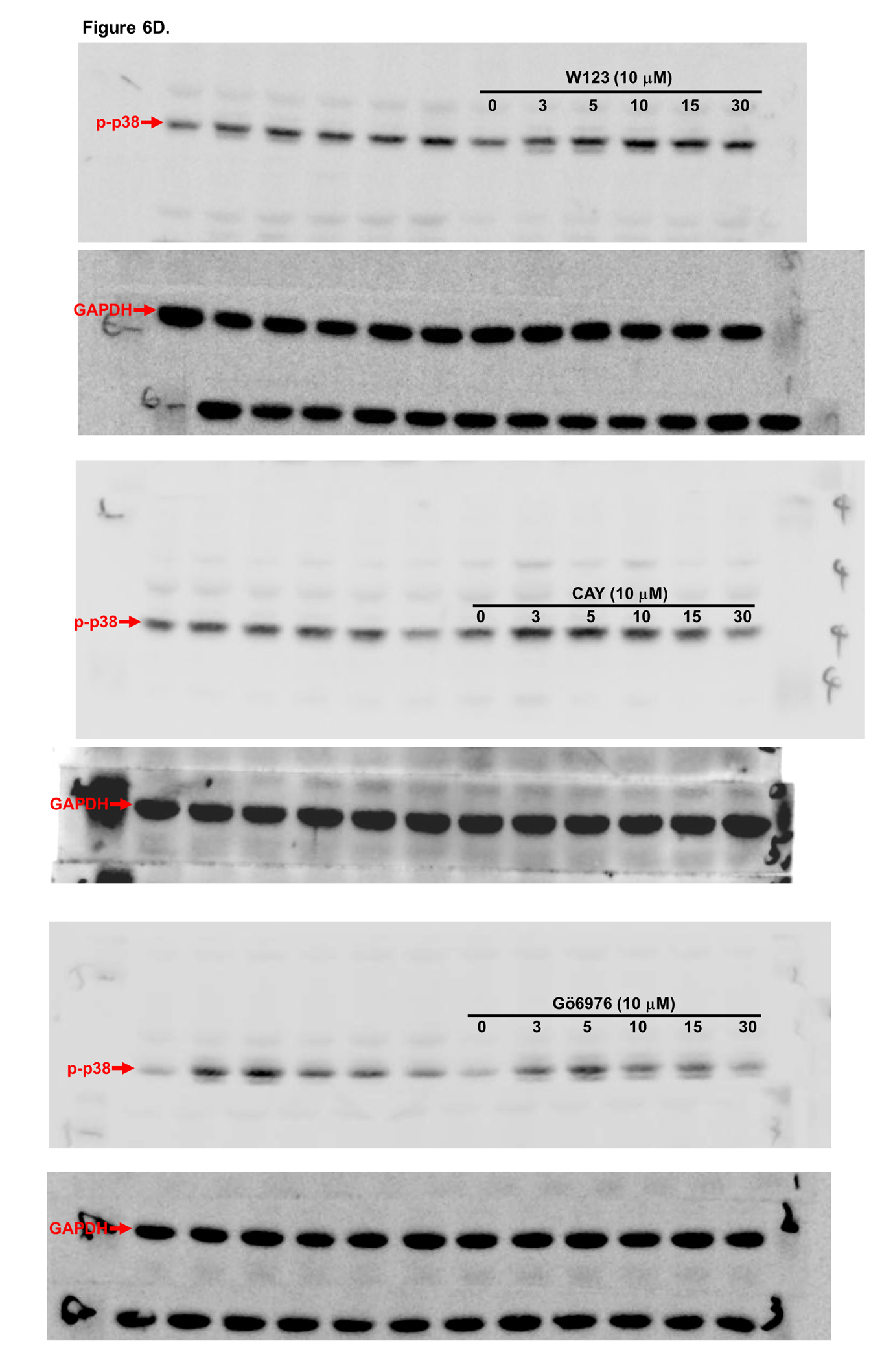

Supplement: Supplementary file 1 [file datasheet1.zip › Supplementary material/Figure 6D-2..tif]

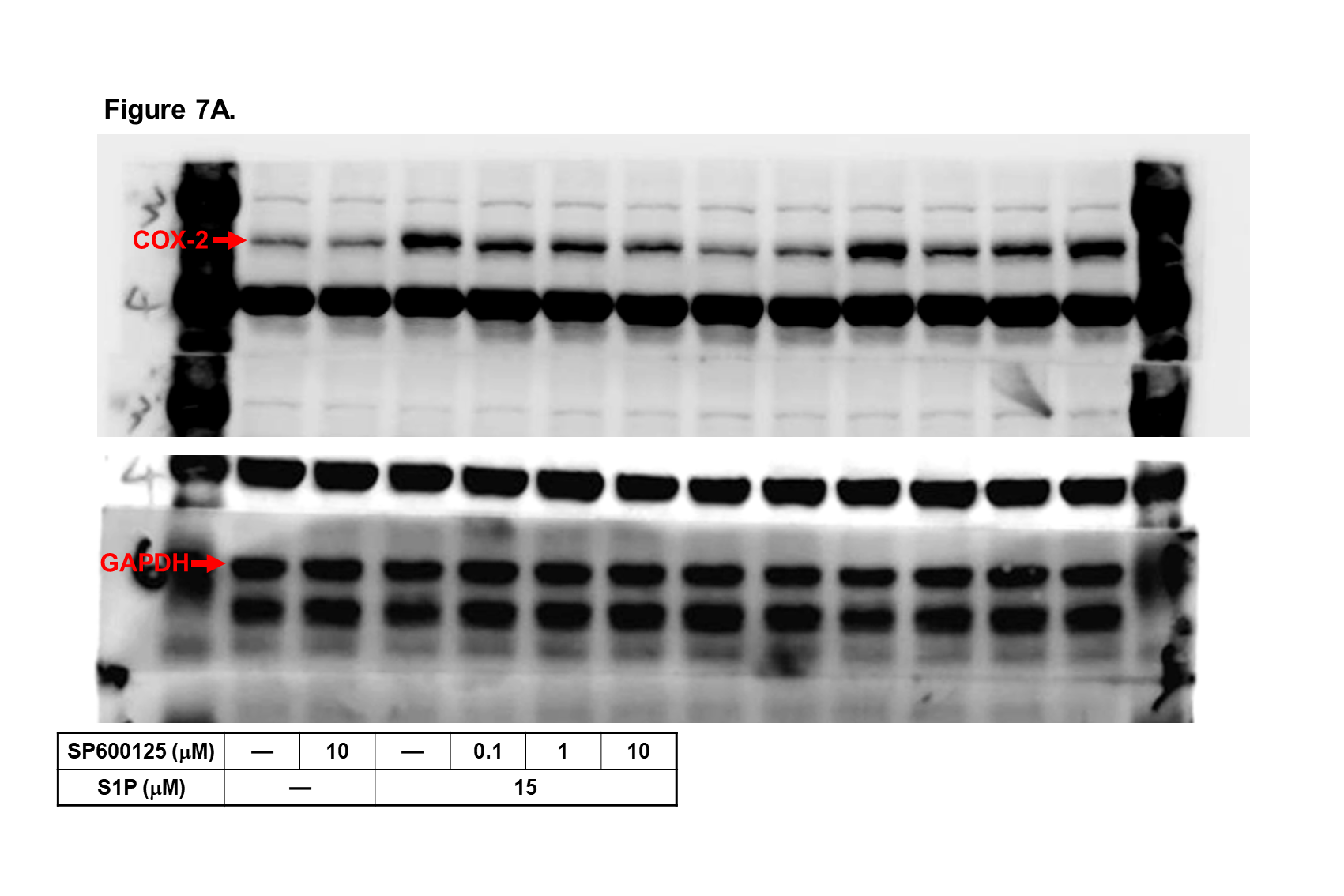

Supplement: Supplementary file 1 [file datasheet1.zip › Supplementary material/Figure 7A..tif]

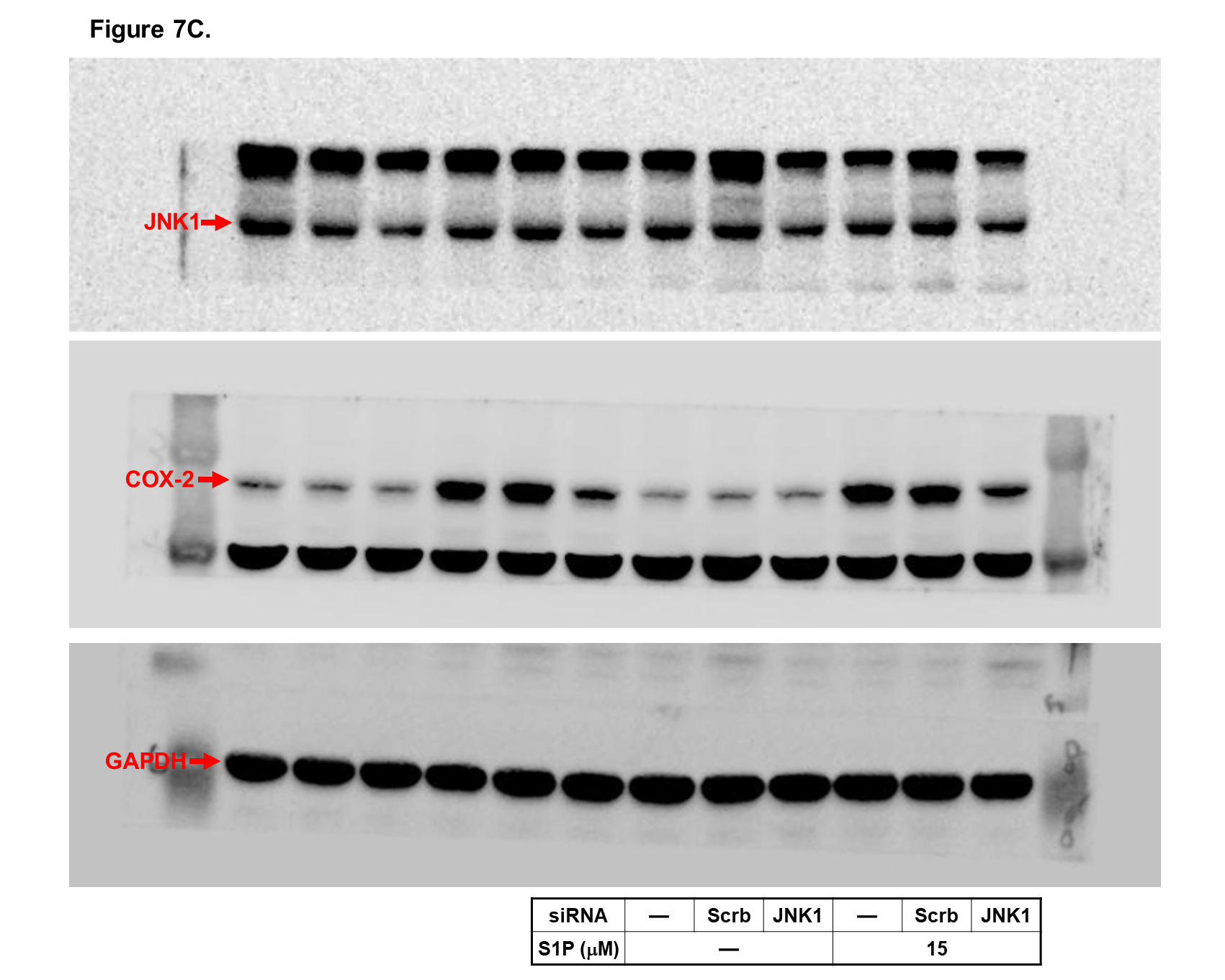

Supplement: Supplementary file 1 [file datasheet1.zip › Supplementary material/Figure 7C..tif]

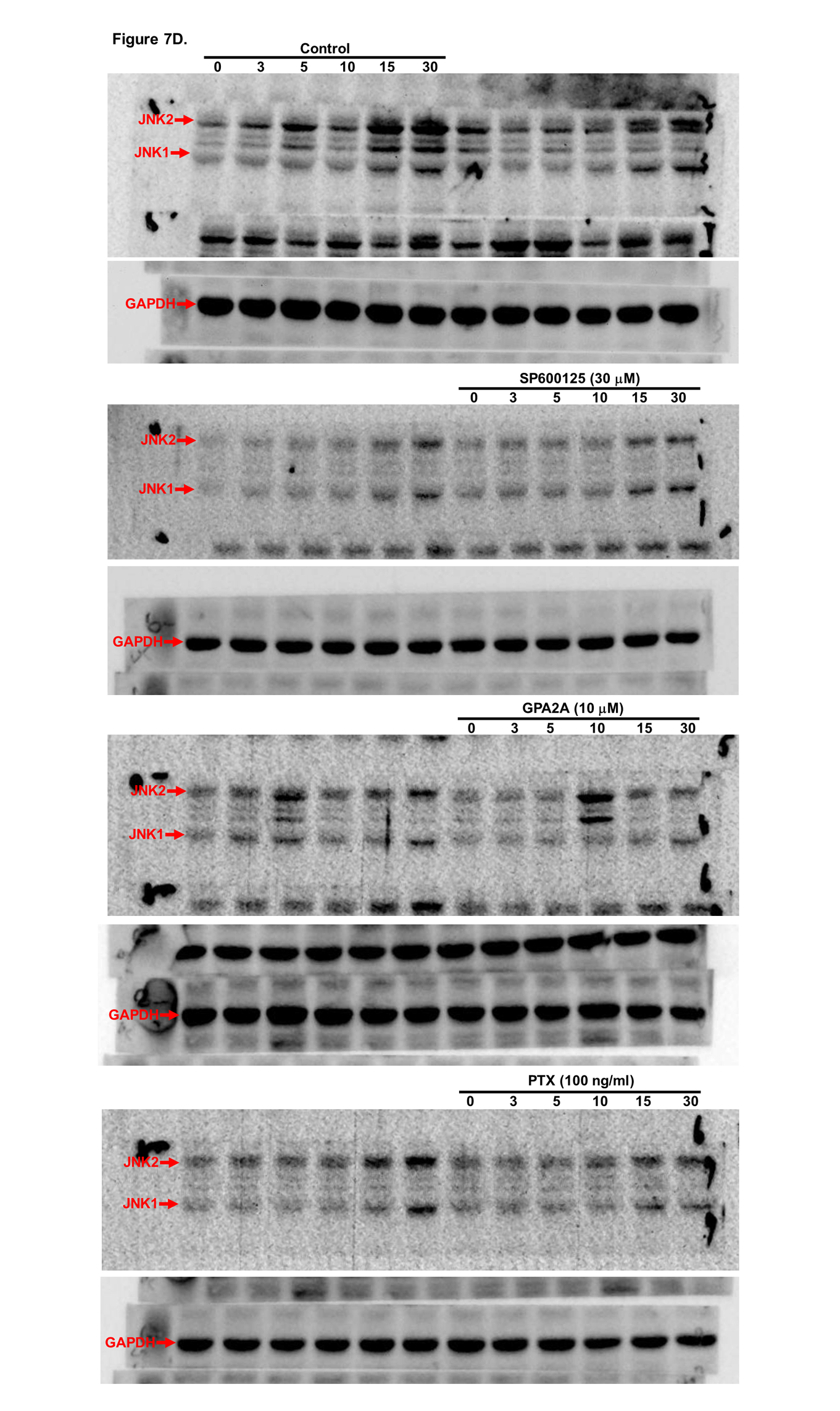

Supplement: Supplementary file 1 [file datasheet1.zip › Supplementary material/Figure 7D-1..tif]

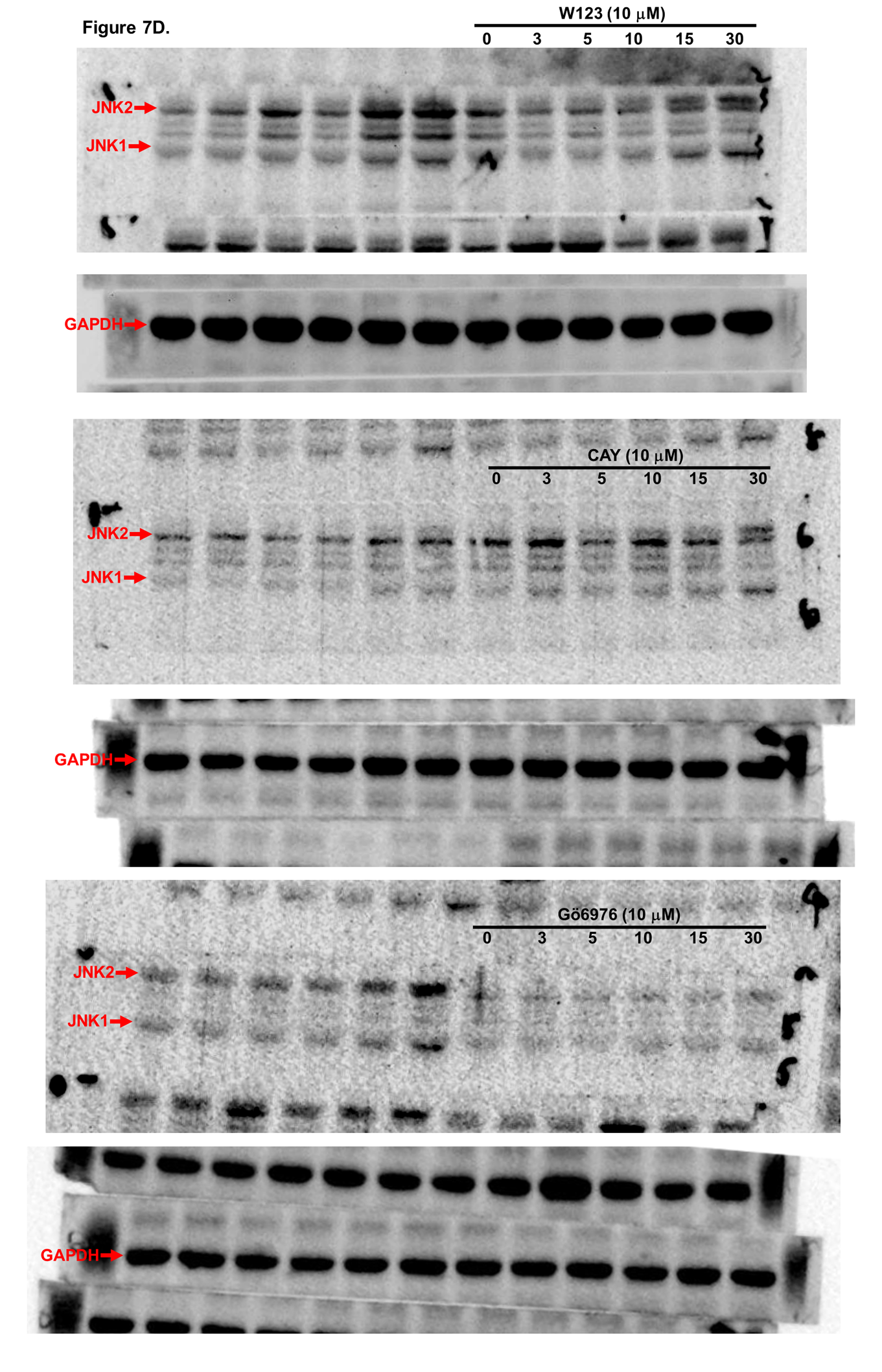

Supplement: Supplementary file 1 [file datasheet1.zip › Supplementary material/Figure 7D-2..tif]

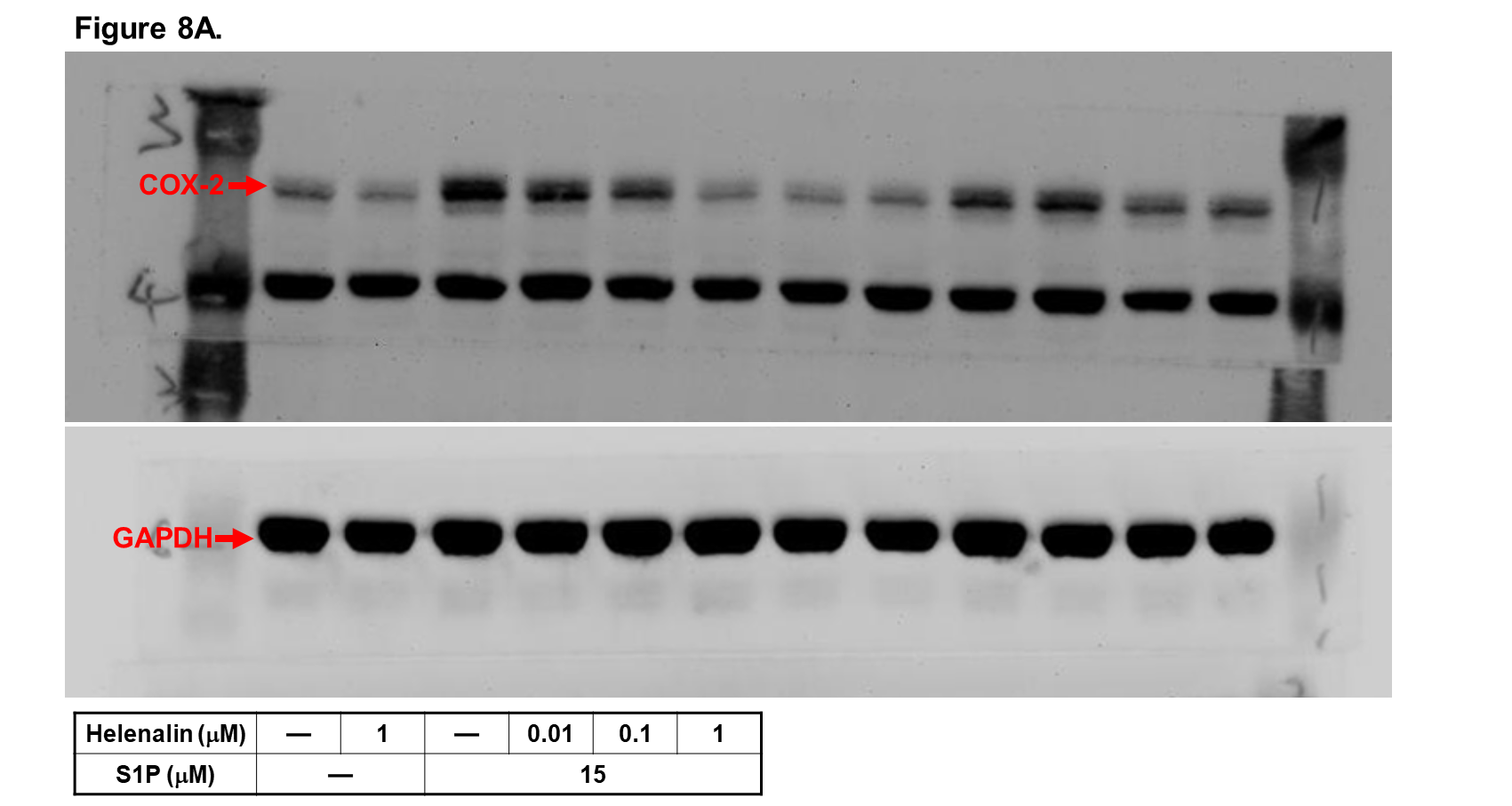

Supplement: Supplementary file 1 [file datasheet1.zip › Supplementary material/Figure 8A..tif]

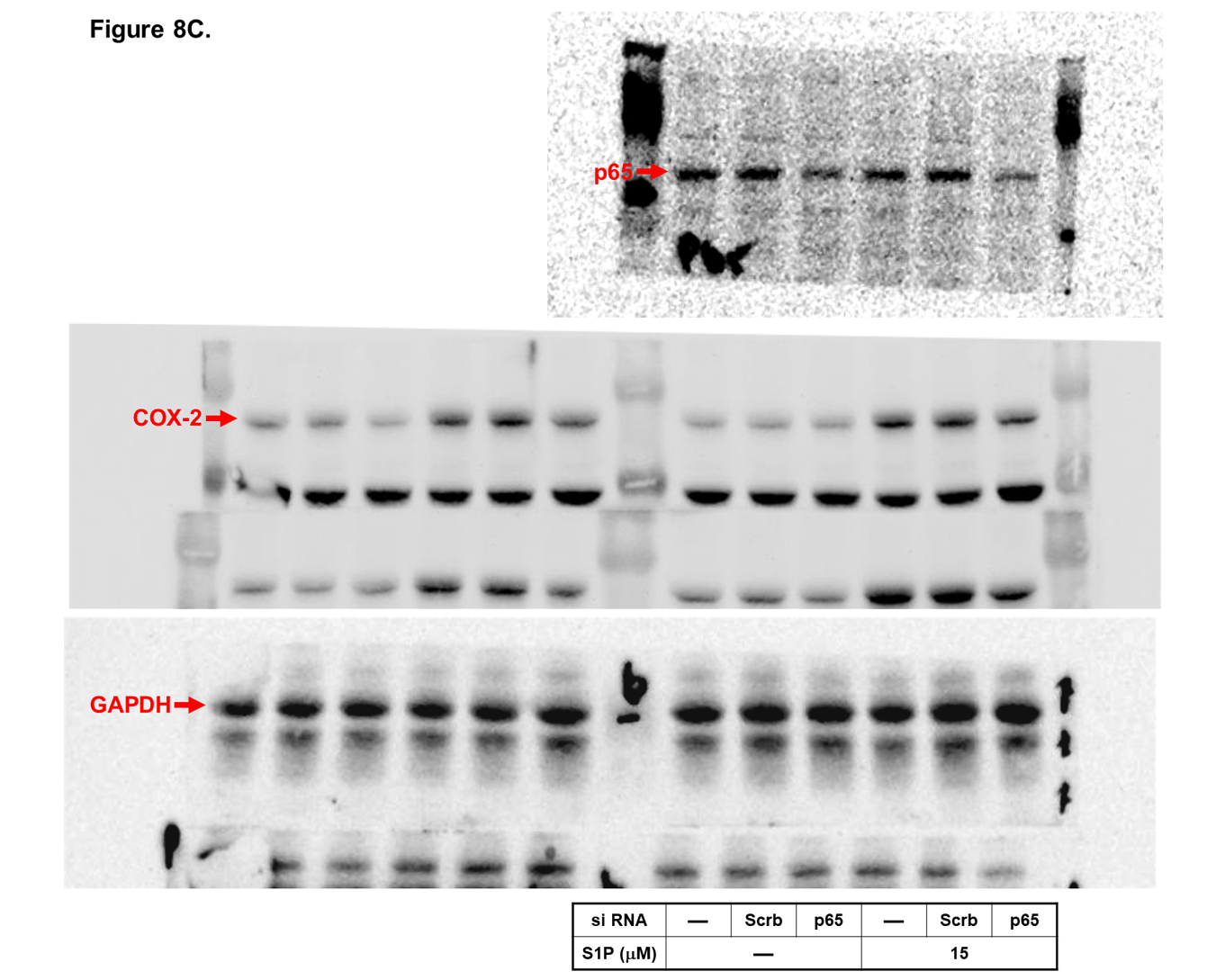

Supplement: Supplementary file 1 [file datasheet1.zip › Supplementary material/Figure 8C..tif]

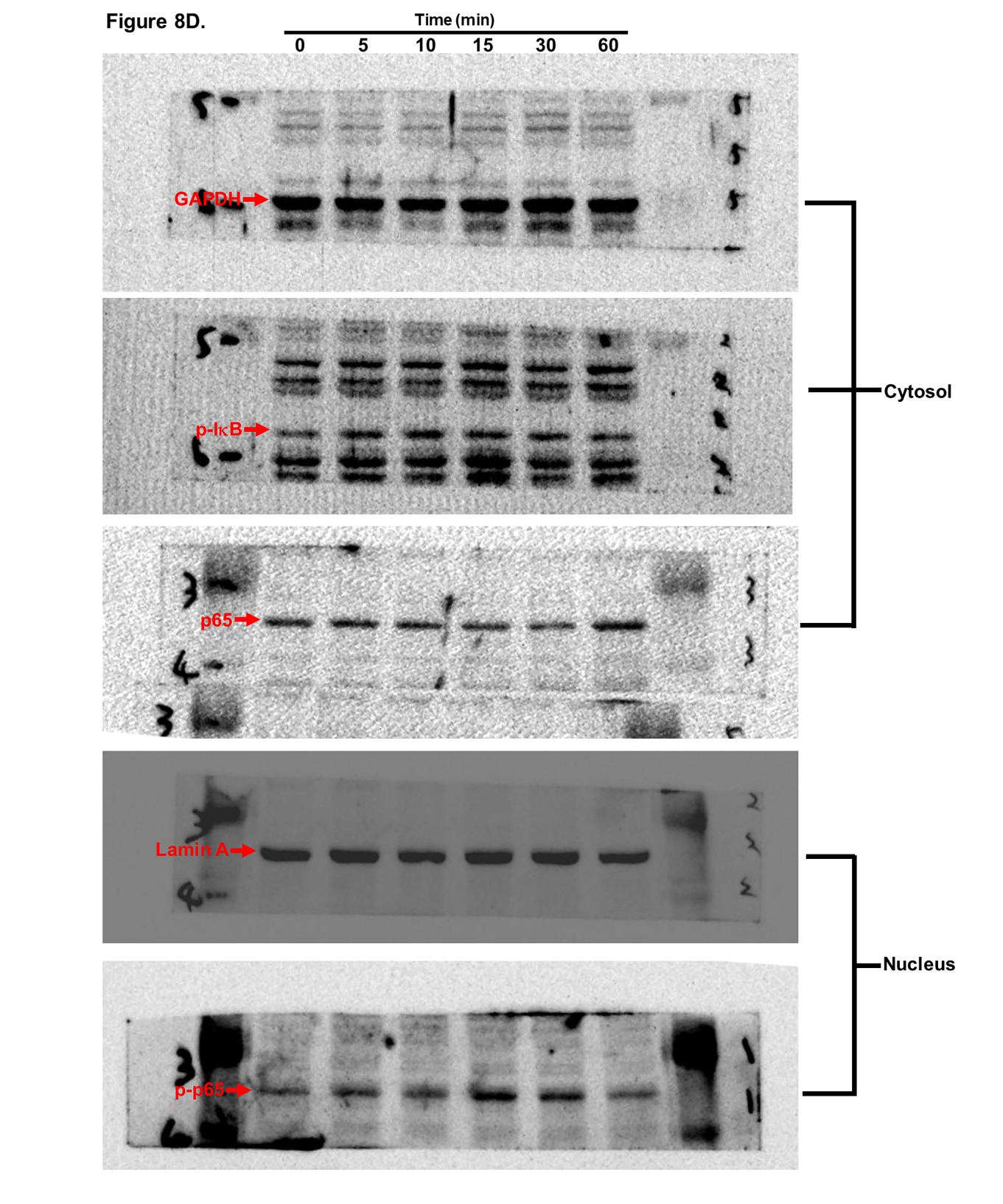

Supplement: Supplementary file 1 [file datasheet1.zip › Supplementary material/Figure 8D..tif]

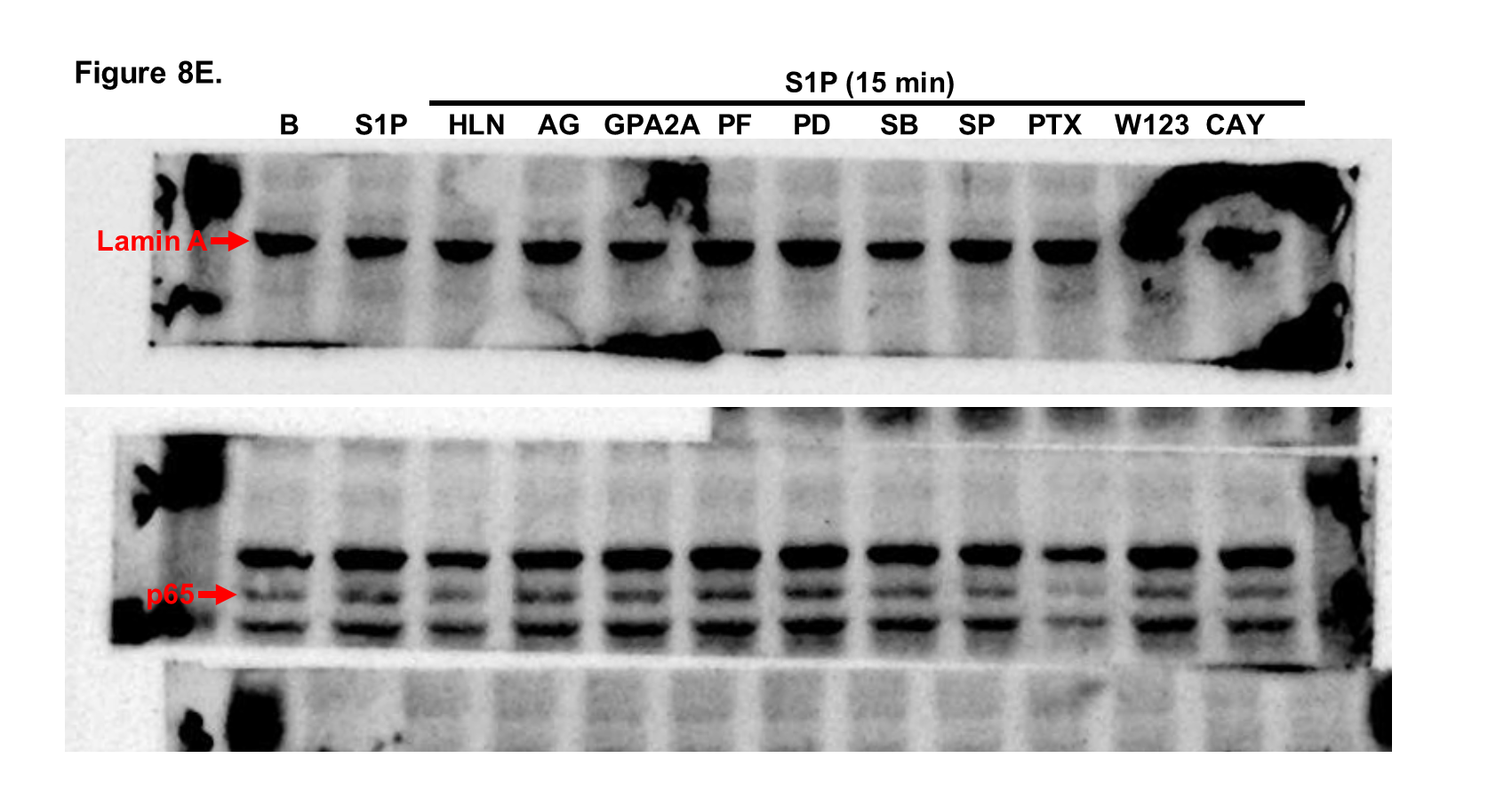

Supplement: Supplementary file 1 [file datasheet1.zip › Supplementary material/Figure 8E..tif]

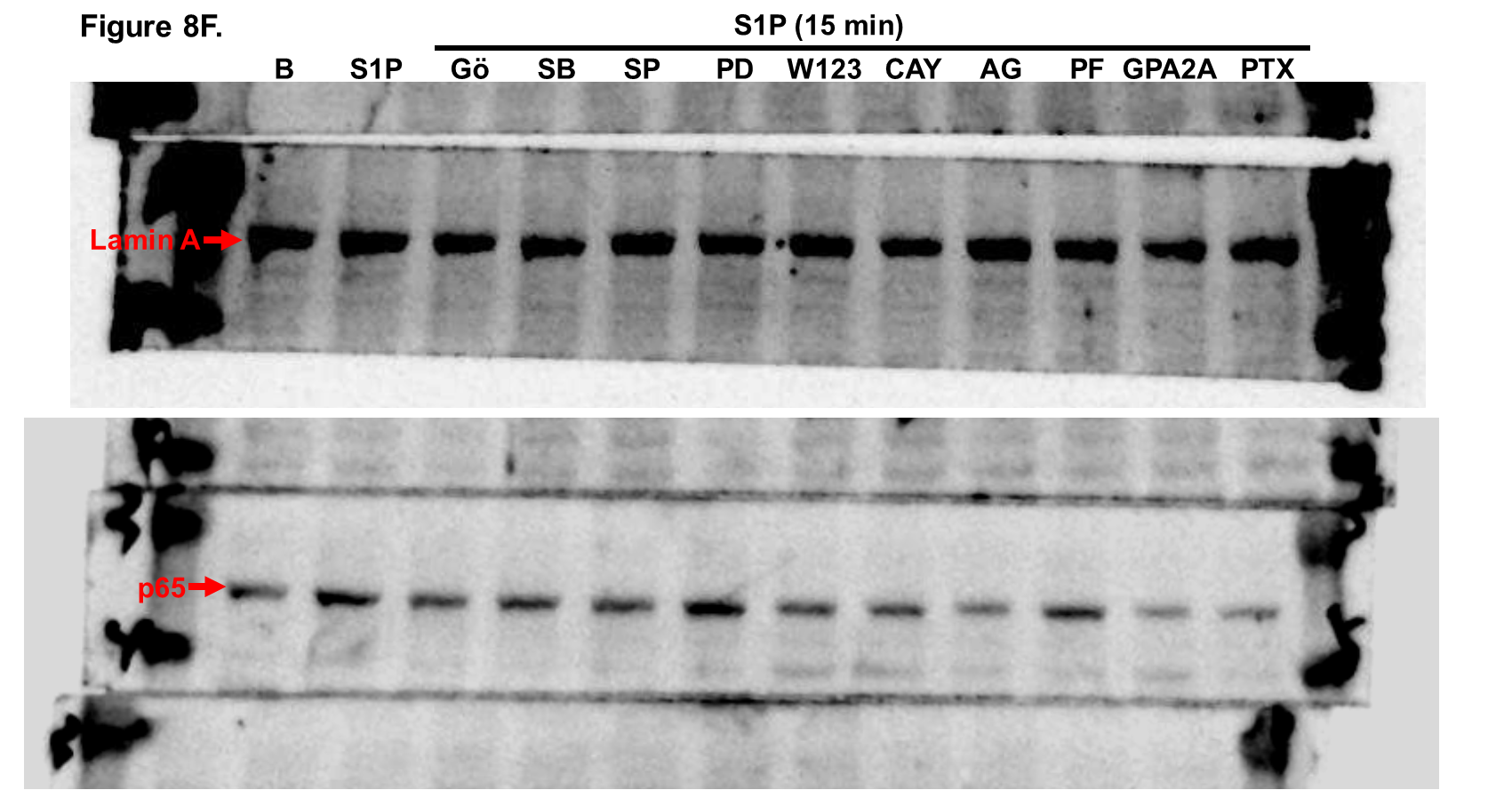

Supplement: Supplementary file 1 [file datasheet1.zip › Supplementary material/Figure 8F..tif]

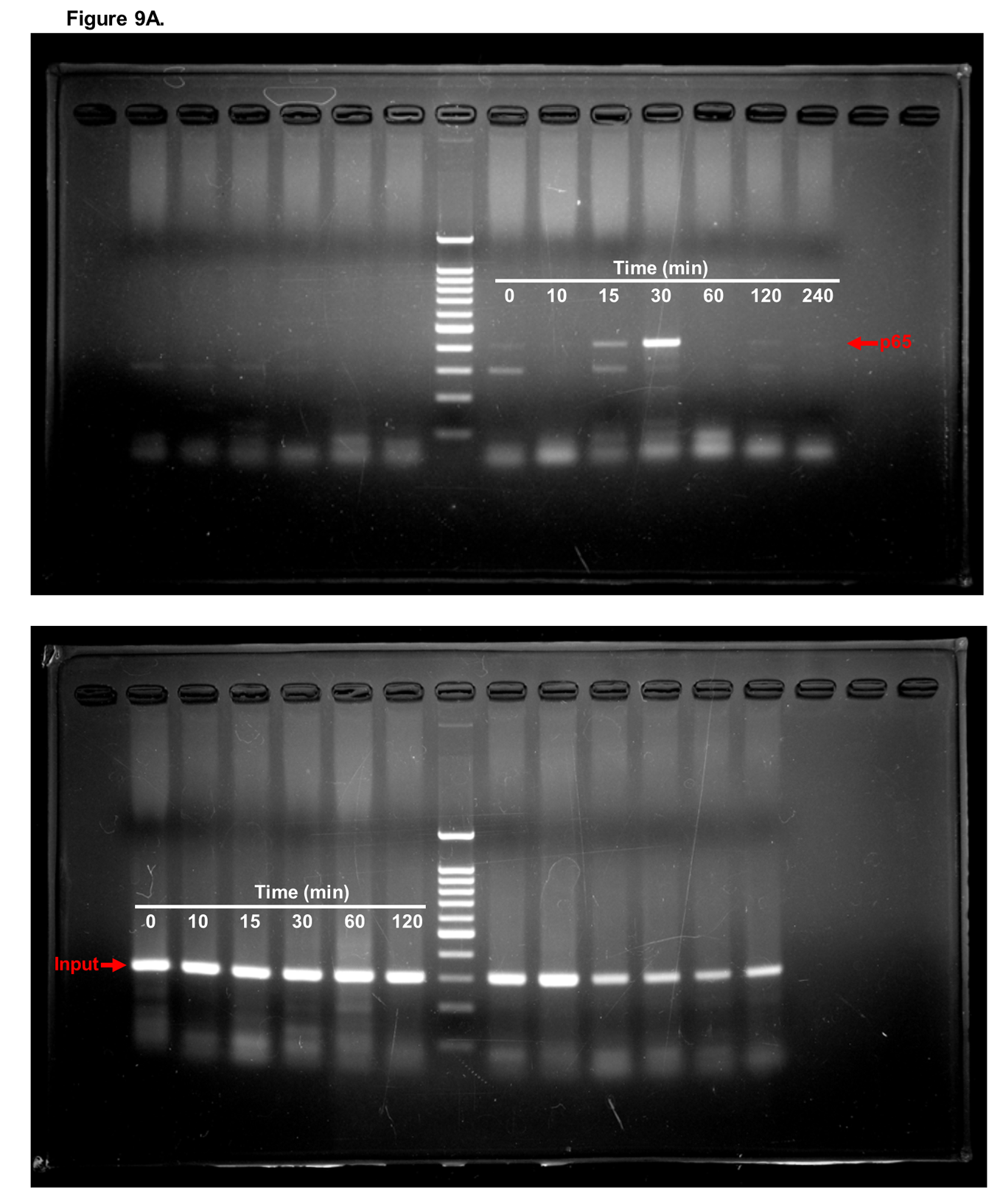

Supplement: Supplementary file 1 [file datasheet1.zip › Supplementary material/Figure 9A-1..tif]

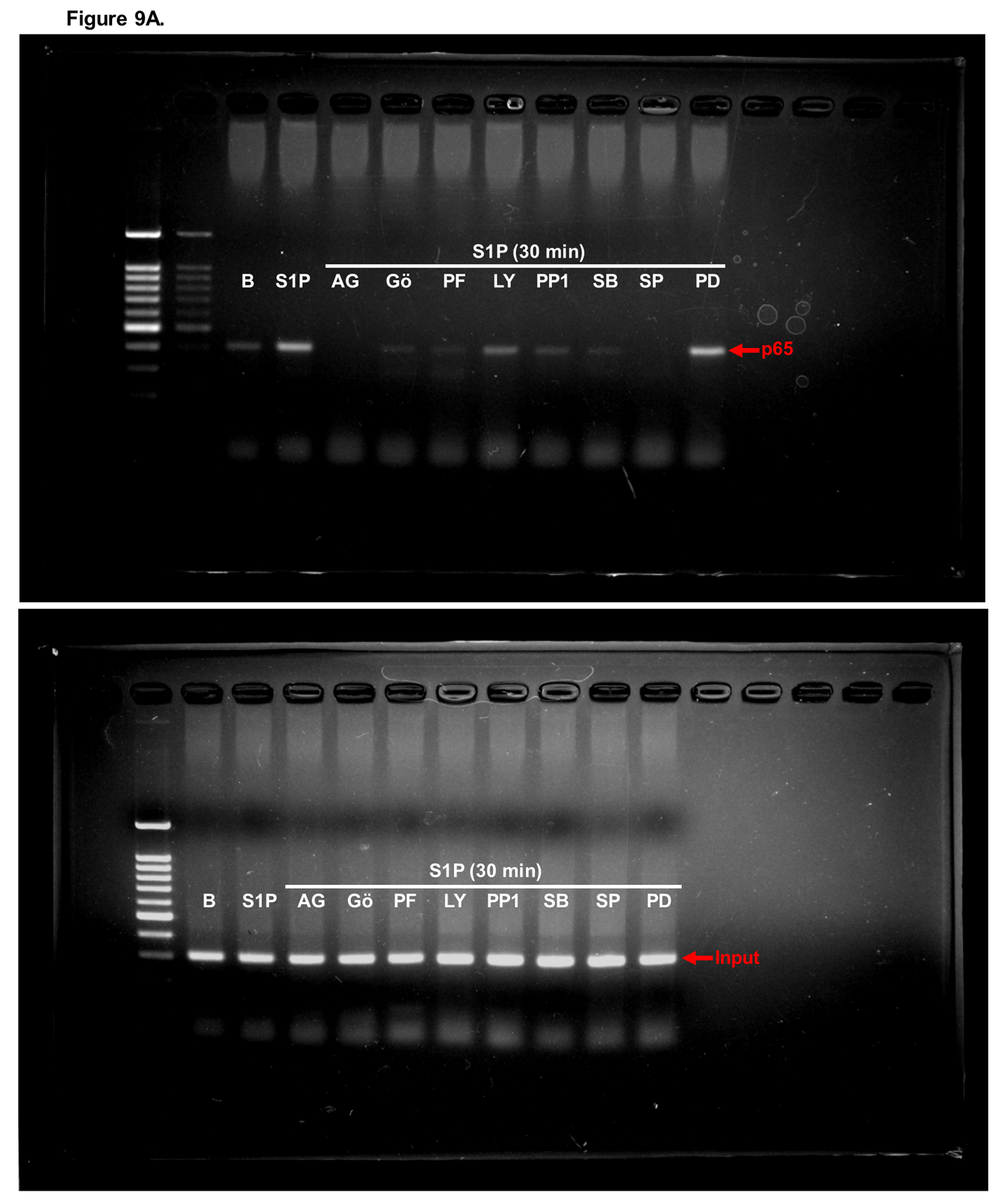

Supplement: Supplementary file 1 [file datasheet1.zip › Supplementary material/Figure 9A-2..tif]
